# Antiaging Properties of the Klotho Protein

**DOI:** 10.3390/cells15060507

**Published:** 2026-03-12

**Authors:** Gérald J. Prud’homme, Qinghua Wang

**Affiliations:** 1 Department of Laboratory Medicine and Pathobiology, University of Toronto, Toronto, ON M5S 1A8, Canada; 2 Department of Laboratory Medicine, Keenan Research Centre for Biomedical Science, Unity Health Toronto, Toronto, ON M5B 1W8, Canada; 3 Department of Endocrinology and Metabolism, Huashan Hospital, Shanghai Medical School, Fudan University, Shanghai 200433, China; 4 Shanghai Innogen Pharmaceutical Co., Ltd., Shanghai 200135, China

**Keywords:** aging, Alzheimer, cancer, FGF23, fibrosis, hallmarks, inflammaging, Klotho, NF-κB, TGF-β

## Abstract

Mice genetically deficient in α-Klotho (henceforth Klotho) display accelerated aging. The mechanisms are only partially understood. Here, we examine how these relate to the 12 hallmarks of aging consisting of chronic inflammation (inflammaging), as well as damaging changes to the genome (DNA damage), telomeres, epigenetic regulation, proteostasis, nutrient sensing, mitochondria, stem cells, intercellular communication, macroautophagy, microbiome and cell replication (senescence). Inflammation aggravates the other hallmarks. We report that Klotho counters the majority of these hallmarks. It ameliorates mitochondrial function and reduces reactive oxygen species (ROS), telomere attrition and cellular senescence. It protects against inflammation by inhibiting NF-κB and the NLRP3 inflammasome. This applies to inflammaging, several chronic inflammatory diseases, atherosclerosis, diabetes, and Alzheimer’s disease. Klotho also counters some aging factors outside of these hallmarks. Low Klotho (often due to kidney disease) produces hyperphosphatemia, which injures cells (especially endothelial cells) and promotes aging. Another key action of Klotho is the mitigation of fibrosis in major organs (kidneys, heart, lungs and other), mainly through the inhibition of TGF-β and Wnt. Klotho also protects against muscle atrophy (sarcopenia)—a common feature of aging—and exhibits anti-cancer activity. We describe several factors that increase Klotho, and are potentially amenable to clinical therapy.

## 1. Introduction

Aging is an inevitable fact of life. It affects all organs and tissues, leading to impaired function, major debilitating diseases and death. Aging can be slowed by lifestyle changes such as exercise and diet. It is accelerated by many well-known negative factors. This includes smoking, obesity, hyperlipidemia, hypertension, chronic infectious diseases, anti-cancer drugs, radiation and exposure to environmental toxins. In diabetic subjects, high or poorly controlled glucose levels markedly accelerate aging. Similarly, renal disease and associated hyperphosphatemia (phosphatopathy) injure cells and accelerate aging [1].

α-Klotho (denoted here Klotho or KL) is an antiaging agent that is expressed in the kidneys, brain, endocrine organs (e.g., pancreatic β cells, parathyroid glands), blood vessels, skin, and other tissues [2]. It is also expressed by peripheral blood cells. Klotho is an obligate coreceptor for fibroblast growth factor 23 (FGF23), an endocrine FGF (eFGF) produced in bone, but it has several other functions, as reviewed by [3]. Klotho-deficient hypomorphic (*KL^kl/kl^*) or null (*KL*^-/-^) mice exhibit rapid aging. This syndrome includes stunted growth, hyperphosphatemia, hypercalcemia, cardiac disease, vascular disease (especially calcification), osteopenia, emphysema, sarcopenia, cognitive deficits, multi-organ atrophy and a short lifespan [4,5]. In contrast, overexpression of Klotho in transgenic mice [6], or induced by gene transfer [7], mitigates several features of aging, and increases lifespan by as much as 20 to 30%. There is a membrane-bound form; as well as a soluble form (s-Klotho) found in the blood and the CSF. In humans, s-Klotho declines with age and in several chronic diseases. Furthermore, low Klotho levels are associated with an increase in mortality. In this review, we focus primarily on the mechanisms by which Klotho delays aging.

## 2. Theories of Aging

There are many theories of aging, but the underlying molecular and cellular mechanisms are still not well defined. Different species age at different rates, suggesting the existence of a poorly understood biological clock [8]. We do not see 80-year old cats or dogs, but many humans surpass that age. The Greenland shark lives hundreds of years. Diverging lifespans (the time from birth to death) most likely represent species evolution in response to the environment, predators, and the availability of food. In order for a species to survive, it has to reproduce at a rate sufficient to match or exceed the death rate. Animals such as mice and rabbits have a short lifespan, but reproduce at a very fast rate. Humans live much longer and reproduce relatively slowly.

Humans are exceedingly complex multi-cellular organisms. Scientists are very far from elucidating all the molecular, cellular and whole organ aspects. To complicate matters, aging affects practically all cells and organs. Different organs can age at a different rate and in different ways. Some connective tissue changes appear irreversible. In view of the biologic complexity, it is not surprising there is no generally accepted theory of aging. Instead, to address this issue, researchers have described a number of hallmarks of aging [9]. There are currently twelve hallmarks, but this could increase. These hallmarks are mostly based on reductionist studies performed in various species (yeast, nematodes, insects and mice), and to a much lesser extent in humans.

### Hallmarks of Aging

These are the twelve hallmarks, as described by Lopez-Otin et al. [9].

Genomic instability/DNA alterations;

Telomere attrition;

Epigenetic alterations;

Loss of proteostasis;

Deregulated nutrient sensing;

Mitochondrial dysfunction;

Cellular senescence;

Stem cell exhaustion;

Altered intercellular communication;

Disabled macroautophagy;

Dysbiosis/altered microbiome;

Chronic inflammation.

**Figure 1:**
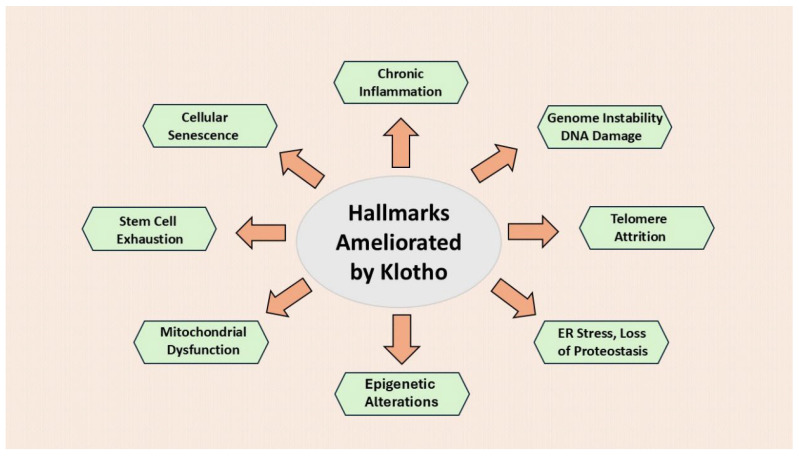
Klotho ameliorates several hallmarks of aging.

**Figure 2:**
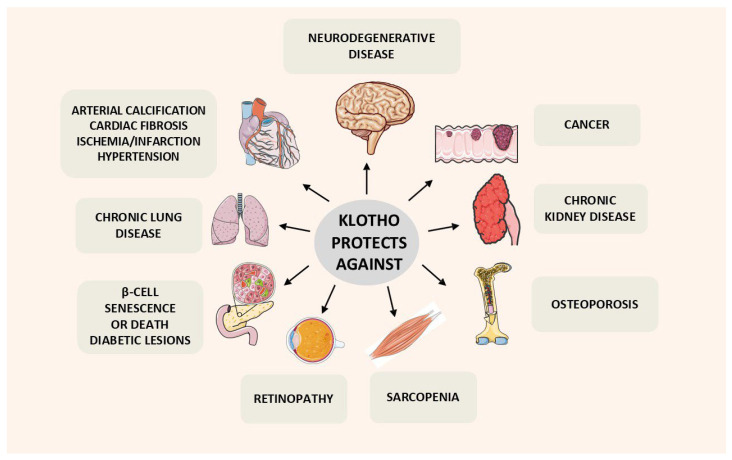
Klotho protects against several pathologies frequently associated with aging.

As outlined in this manuscript, Klotho mitigates several of these hallmarks (Figure 1). The description of aging hallmarks has been very useful for research, but it has limitations. Aging and the specific causes of death differ markedly between species. The relative importance of each hallmark is not well defined. They are based on important general concepts, but do not specifically address diseases. For example, cell death and the importance of various pathologies are not part of this scheme. Klotho ameliorates several age-related factors and pathologies (Figure 2). However, in many of these cases the actual mode of action of Klotho has not been established.

Autopsy studies in human populations provide important information. In order to age, an individual has to survive (obviously). A single organ failure frequently leads to death, regardless of the health of the other organs. For example, a person who appears to be in perfect health can die suddenly from a short thrombotic obstruction of a 3 or 4 mm diameter coronary artery. A similar occlusion of the middle cerebral artery can cause a massive fatal stroke. Uncontrolled type 2 diabetes potentially leads to fatal outcomes. These disease conditions are not generally considered “aging,” but occur at a much higher frequency with age. In effect, people do not die from old age, but rather from diseases that are much more common in the elderly.

Pathologies such as atherosclerosis, thrombosis, infarction, cancer, diabetes, fibrosis, emphysema, end-stage renal failure, neurogenerative diseases, autoimmune diseases, chronic inflammatory diseases, infections (deficient immunity), etc., all reduce life expectancy. These conditions are generally multi-factorial and complex. By far, in industrialized countries, death is due to either vascular disease (especially myocardial infarcts and strokes) or cancer in its many forms. Consequently, therapies or lifestyle changes that reduce vascular disease and cancer will have a major impact. Chronic lung diseases, Alzheimer’s disease and diabetes are also common causes of morbidity and mortality. Remarkably, numerous preclinical studies have shown that Klotho has protective effects against most of these major diseases.

## 3. Overview of Antiaging Activities of Klotho

As mentioned previously, Klotho action mitigates several hallmarks of aging (Figure 1), and ameliorates numerous age-related pathologies (Figure 2). It is beyond the scope of this manuscript to cover all these aspects in detail. However, concerning the pathobiology of Klotho, there have been a number of relevant reviews published by others and us [3,5,10,11,12,13,14,15,16,17]. Here, we list the Klotho activities that appear most relevant to aging, whether or not they are linked to the 12 hallmarks. These findings were mostly obtained from preclinical work, but they are in accord with available clinical findings. This is a brief overview of these findings, and several of these features are discussed in subsequent sections of the manuscript.

(1) **Regulation of phosphate/calcium homeostasis.** Klotho deficiency, often due to kidney disease, causes hyperphosphatemia and associated pathogenic calciprotein particles (CPPs). It has been proposed that this plays an important role in the pathophysiology of aging [1,5,13,14]. Phosphate overload (phosphatopathy) induces inflammation, oxidative stress, endothelial injury, vascular calcification, cellular senescence, Wnt/β-catenin signaling, TGF-β action and other anomalies [1].
(2) **Reduction in DNA damage, telomere attrition and stem cell depletion.** Klotho protects against these three major aging factors [18,19,20,21]. The mechanisms are not well delineated; however, the inhibition of Wnt ligands mitigates stem cell loss [22].
(3) **Reduction in cellular senescence and mitochondrial dysfunction.** Senescence and disordered mitochondrial function are well recognized features of aging, and are both ameliorated by Klotho [23,24,25,26,27].
(4) **Protection against inflammaging and chronic inflammatory pathologies.** This is a prominent action of Klotho [16]. Inflammation aggravates most of the hallmarks of aging, including genomic instability, telomere attrition, epigenetic alterations, proteostasis, macroautophagy, deregulated nutrient sensing, mitochondrial dysfunction, cellular senescence and stem cell function [9,28]. In addition, inflammation can cause cell death by necrosis, apoptosis, pyroptosis and necroptosis.
(5) **Reduction in endothelial-cell injury.** Endothelial injury promotes age-related pathologies [23,29,30]. It contributes to early vascular aging in chronic renal disease, diabetes, hypertension and cigarette smoking.
(6) **Reduction in endoplasmic reticulum (ER) stress.** Klotho mitigates ER stress and the unfolding protein response [31,32]. Excessive ER stress contributes to dysfunctional proteostasis, the accumulation of pathogenic protein aggregates and cell death; for example, in neurodegenerative diseases [33,34].
(7) **Depletion of reactive oxygen species (ROS).** Excessive ROS production (oxidative stress) is widely considered a major cause of aging. Klotho improves mitochondrial function, which reduces ROS [35,36,37,38]. Furthermore, it induces Nrf2 and FoxO which increase antioxidant enzymes that eliminate ROS.
(8) **Prevention of organ fibrosis.** Klotho reduces fibrosis in major organs (e.g., kidneys, heart, lungs and liver), mainly through inhibition of TGF-β and Wnt [39,40]. Fibrosis often follows inflammation, and at advanced stages can lead to organ failure.
(9) **Mitigation of sarcopenia.** Prevention of muscular atrophy (sarcopenia) through inhibition of myostatin, TGF-β and other TGF-β family members [41], as well as anti-inflammatory and other factors [42]. Sarcopenia is a common and debilitating factor in aging, cancer and many chronic diseases.
(10) **Tumor suppressor functions.** This is likely related to inhibition of multiple pathways such as insulin-like growth factor 1 (IGF-1), TGF-β, Wnt and NF-κB, as well as enhancement of epigenetic regulation [15,43].
(11) **Delayed aging as determined by biomarkers.** When examined with DNAm-Phenoage, a DNA methylation epigenetic aging clock, higher Klotho levels appear to delay human aging [44]. This assay is predictive of all-cause mortality and age-related pathologies. The mechanisms of Klotho action are likely complex. For instance, a preclinical study revealed that overexpression of Klotho altered the expression of all the groups of genes associated with the hallmarks of aging [45].
(12) **Reduced mortality rate.** A low circulating Klotho level is a marker for an increased risk of death; either all-cause or in subjects with cardiovascular disease, renal disease, cancer, old age and other conditions [46,47,48,49,50,51].

## 4. Molecular Features

The molecular aspects of Klotho and its function as a coreceptor for FGF23 have been extensively studied, as reported by several researchers [5,10,52,53,54,55,56,57,58,59,60,61,62,63]. Klotho is expressed on the membrane as a single-pass protein, with a very short cytoplasmic segment (10 aa). In humans it has 1012 aa (130 kD). It has two extracellular domains of similar size—denoted KL1 and KL2. A soluble form of Klotho (s-Klotho) is produced by shedding. This soluble form is also termed processed Klotho (p-KL) by some authors [59]. It is generated by the cleaving action of extracellular proteases, principally ADAM10 and ADAM17 (α-secretases) [58,60]. The released KL1/KL2 segment appears to be the main (and perhaps only) circulating form of Klotho. This is consistent, for example, with the depletion of circulating Klotho observed in mice treated with secretase inhibitors.

However, a smaller form generated by alternative splicing has been described. It encodes KL1 and an additional C-terminal tail of 14 amino acids (but not KL2) [59]. It is termed secreted Klotho (sometimes confusingly abbreviated s-KL). Its existence has been questioned by some authors [61]. These researchers reported that the alternative *Klotho* mRNA (putative secreted Klotho) has premature termination codons that mark it for degradation by nonsense-mediated RNA decay (NMD). They could not identify production of the protein in vitro. To our knowledge there has been no other publication about *Klotho* NMD. Interestingly, unlike mice and humans, rats are devoid of this alternative splice variant. In any event, the secreted form of Klotho can be produced by gene therapy vectors, which presumably carry only the coding sequence (no RNA splicing) [7,59]. This allows selective expression of the secreted KL1 domain in vivo, and this has been linked to increased longevity and health in mice [7].

Crystal structure analysis demonstrated that Klotho binds to FGF receptors to produce a high affinity receptor for FGF23 [53]. In the absence of Klotho, FGF23 has low affinity for FGFRs. Klotho has affinity for FGFRs 1c, 3c and 4 [53,62], although FGFR1c appears to be employed the most often. Klotho attaches to FGFR1c by an extension of its KL2 domain. FGF23 binds into a groove formed by components of KL1, KL2 and the FGFR [53,63]. Thus, KL2 is required for receptor assembly and FGF23 binding. The KL1 domain also contributes to FGF23 binding, but does not interact directly to FGFRs.

Importantly, KL1 (with or without KL2) performs many Klotho functions that appear FGF23-independent. This includes blockade of TGF-β, Wnt ligands, insulin-like growth factor 1 (IGF-1) and other mediators. For example, a KL1 domain construct exerted anti-cancer activity, but did not promote FGF23 signaling [64]. Related to this issue, Roig-Soriano et al. [59] compared in vivo gene transfer-based treatment with processed Klotho (KL1/KL2 segment) versus secreted Klotho (KL1 domain only). The processed Klotho differed markedly in that it increased FGF23 production in bone, and altered bone microstructure. It also perturbed phosphate and calcium homeostasis. The secreted Klotho did not have these effects. This is consistent with soluble KL1/KL2 acting as a FGF23 coreceptor, although this question was not examined.

In accord with in vitro and in vivo studies, s-Klotho (soluble KL1/KL2) can attach to FGFRs, and function as a coreceptor for FGF23 [53,63,65,66]. This is supported by the finding that s-Klotho protein injection into *Klotho* hypomorphic mice increased urinary phosphate excretion [67]. In wild-type mice, s-Klotho similarly increased phosphate excretion and lowered serum phosphate [63]. In vitro, s-Klotho enhanced FGF23-induced signaling in Klotho-negative HEK293 cells [53]. Co-stimulation with soluble Klotho appears weaker than with membrane Klotho. Of note, free FGF23 can also bind directly to free s-Klotho (KL1/KL2) at relatively high affinity [56,57,68]. Presumably, the FGF23/s-Klotho soluble complex can then attach to membrane-bound FGFRs and induce signaling. However, the extent to which s-Klotho promotes FGF23 signaling under ordinary physiological conditions remains unclear.

## 5. Renal Physiology

Klotho physiology in the kidney is the area that has been the best studied and characterized [5,54,55,69,70,71]. Upon binding of both Klotho and FGF23, the activated FGFR signals through multiple pathways, notably PI3K/Akt, Ras/MAPK/ERK and phospholipase Cγ (PLCγ). The activated FGFR1c receptor contributes to phosphate and calcium exchange. In the proximal renal tubule, inorganic phosphate (Pi) re-absorption is reduced (phosphaturic effect), through inhibition of the sodium-phosphate transporters NPT-2a and NPT-2c [53]. Klotho’s action in the distal tubule is different. There, Ca^2+^ resorption is enhanced by TRPV5 channels. Of importance, FGF23/Klotho/FGFR1c regulates vitamin D by inhibiting 1α-hydroxylase in the proximal renal tubule. This reduces the synthesis of active vitamin D (1,25(OH)_2_D_3_)—termed calcitriol. There is also increased enzymatic inactivation of calcitriol, but the regulation of this is not totally resolved [71].

## 6. Human Mutations

Lack of either Klotho or FGF23 in mice results in hyperphosphatemia, hypercalcemia, 1α-hydroxylase overactivity and hypervitaminosis D. A low phosphate diet improves disease, despite the fact that vitamin D increases further [5]. In humans, Klotho deficiency is most commonly the result of acute or chronic kidney failure [55]. Note that contrary to *Klotho* hypomorphic mice, these persons usually have low vitamin D levels. This is consistent with advanced renal disease.

Rare human mutations (usually autosomal recessive) cause a severe loss of either FGF23 or Klotho [5,72,73]. Nearly all cases involve *FGF23*. Very infrequently, low FGF23 levels are caused by anti-FGF23 antibodies, in an autoimmune process. To our knowledge, a *Klotho* loss of function mutation has only been reported in one person [74]. In all these different cases, the subjects have hyperphosphatemia. Vitamin D levels may also be high. At a young age, these patients develop massive calcification in soft tissues (tumoral calcinosis); as well as calcification of the arteries, and calcification in multiple organs and tissues. They have bone and dental anomalies, and systemic inflammation. To our knowledge, there has not a been a detailed analysis of aging over a long period of time in these individuals. However, hyperphosphatemia and systemic inflammation are factors that can accelerate aging. Note that subjects with FGF23 deficiency can still produce Klotho, which has FGF23-independent functions. In usual end-stage renal disease Klotho levels are low, and these persons have hyperphosphatemia and multiple other anomalies [5,55].

Some clinical FGF23 overproduction syndromes have been reported. These are due to mutations that increase FGF23 levels, or to FGF23-secreting tumors [73,75]. As expected, these individuals develop hypophosphatemia. Similarly, a translocation causing excessive Klotho production (single case reported) was associated with hypophosphatemia [76].

In addition to these findings, several *Klotho* genetic variants have been described [77,78,79,80,81,82]. The best characterized is denoted KL-VS haplotype. It consists of six single nucleotide polymorphisms (SNP) in strict linkage disequilibrium [77]. The KL-VS haplotype carries two mutations in the coding sequence that produce amino acid substitutions (F352V and C370S). Its occurrence varies in different populations, and it is present in 15% of Caucasians. In neuropathologies and other conditions it is generally protective in the heterozygous state, rather than in the rarer homozygous state where it can be detrimental. KL-VS heterozygosity associates with increased lifespan [78,80]. It has been linked to improved cognitive functions, as well as reduced amyloid-β (Aβ) and risk of Alzheimer’s disease [77,79,80]. Notably, KL-VS heterozygosity is associated with reduced neuroinflammation and neurodegeneration, at least in some of the subgroups that were examined [79]. KL-VS heterozygosity also appears to be protective against cardiovascular disease [81]. Several other *Klotho* polymorphisms have been associated with disease, but have not been studied as thoroughly as KL-VS. For example, some *Klotho* genetic variants altered disease expression in either a positive or negative way as related to cognition [77] and diabetes [82]. A caveat is that the beneficial effects of KL-VS heterozygosity have not been confirmed in all studies. The role of the other variants requires more extensive investigation. In all cases, the protective mode of action is not well elucidated.

## 7. Anti-Inflammatory Activities of Klotho

### 7.1. Inflammaging

The ability of Klotho to suppress inflammation is possibly its most important function, as related to aging [16,17]. Acute inflammation occurs in numerous settings; very often against infectious agents or in response to tissue injury (heat, toxins, drugs, ischemic necrosis, radiation, etc.). However, various self-components can initiate a type of sterile chronic inflammation associated with aging, and often denoted inflammaging [9,16,28]. Chronic low-grade inflammation is stimulated by pathological products or deposits such as β-amyloid (Alzheimer’s disease), calcium deposits (hypercalcemia/hyperphosphatemia), advanced-glycation end products (AGEs; hyperglycemia/diabetes), dead cell components, and many other factors. As mentioned previously, inflammation exacerbates practically all of the 12 hallmarks of aging [9,28]. Furthermore, it can initiate fibrosis in most tissues, and precipitate organ failure. Thus, the suppression of inflammation as performed by Klotho likely mitigates many aspects of aging.

### 7.2. Negative Regulation of the NF-κB Pathway

The inhibition of NF-κB by Klotho is an anti-inflammatory mechanism that has been often reported, as we reviewed in [16]. NF-κB is present in immune cells, and practically all other cell types. It plays a major role in initiating inflammatory and/or immune responses in all branches of the immune system [83,84]. NF-κB can be activated by several mechanisms of both innate and adaptive immunity. This includes receptors for damage-associated molecular patterns (DAMPs) and pathogen-associated molecular patterns (PAMPs), such as toll-like receptors (TLR) and NOD-like receptors (NLR). Activation can also be mediated by many other stimuli such as lymphocyte costimulatory molecules, antigen receptors, cytokines, chemokines, and stimulator of interferon genes (STING). Furthermore, NF-κB intersects with the coagulation system to induce both inflammation and thrombosis [83].

NF-κB is not a single protein but rather a family of transcriptional factors [83,84]. The key components involved are p105 (the precursor of p50), p100 (the precursor of p52), p65 (RelA), RelB, and REL (c-Rel). There are two main activation pathways, termed canonical and alternative (noncanonical), but most cells employ the canonical pathway. NF-κB signaling is initiated by the activation of kinases, such as TAK1 and the IKK complex (NEMO/IKKα/IKKβ). In resting cells, the inhibitor of κB (IκB) is bound to NF-κB (p50/p65), which blocks activation. In response to a triggering event, the IκB protein is degraded. This releases NF-κB which then migrates into the nucleus, binds to DNA, and promotes the transcription of hundreds of genes.

In several studies, Klotho blocked NF-κB nuclear translocation, or other NF-κB activating events, in endothelial cells, macrophages, pancreatic β cells and other cells [30,85,86,87,88,89,90,91,92,93,94,95]. Importantly, in cells that lacked Klotho, the addition of s-Klotho to cultures generated Klotho action. This suggests that s-Klotho was endocytosed, but the mechanism has not been elucidated.

### 7.3. Klotho Suppresses Activation of the NOD-like Receptor Pyrin Domain Containing 3 (NLRP3) Inflammasome

Inflammasomes are large cytoplasmic molecular complexes that detect a variety of injurious (danger) signals. Once activated, the inflammasome activates caspase-1 (or other caspase). The caspase cleaves and activates IL-1β and IL-18, as well as a protein termed gasdermin D. The cleaved gasdermin forms a pore on the membrane, which releases IL-1β and IL-18. But, the pores also provoke lytic cell death (pyroptosis) [96,97]. Several inflammasomes have been characterized (e.g., pyrin, NLRP1, NLRP3, NLRC4, AIM2), and each responds to a different set of danger signals [96,97,98]. Inflammasomes are in immune cells and numerous other cell types.

The NLRP3 inflammasome is the best characterized, and it is triggered by many more stimuli than other inflammasomes [19,96,97,98,99]. The activation of the NLRP3 inflammasome depends on two signals [100]. The first signal is mediated by NF-κB, and it increases the intra-cellular levels of the proteins required for inflammasome assembly. The activation event (signal 2) induces inflammasome assembly and function by mechanisms that are not completely elucidated. It can be delivered by several potential stimuli such as ion fluxes (especially K^+^ efflux), reactive oxygen species (ROS), crystals (e.g., urate), calcium deposits, lysosomal rupture, components released by dead cells (e.g., ATP), PAMPs, amyloid-β, AGEs, and other mechanisms [97,98,99,100,101]. Klotho blocks signal 1, and in many circumstances mitigates signal 2.

Of particular interest, Klotho inhibited the NLRP3 inflammasome in endothelial cells [29]. In this case, secreted IL-1β (from the inflammasome) bound to IL-1 receptor of endothelial. This stimulated NF-κB and NLRP3 inflammasome activation in a positive feedback loop (autoactivation). Autoactivation was blocked by Klotho, which prevented endothelial dysfunction.

The anti-inflammatory functions of Klotho are relevant to the treatment of sepsis [102,103]. Sepsis is frequently associated with a severe systemic inflammatory response (septic shock), multiple-organ failure, and a high rate of mortality. Klotho deficiency aggravates the severity of sepsis. This might explain why aged individuals have a reduced resistance to sepsis. In preclinical studies, Klotho administration exerted protective effects against endotoxemia in mice (a model of sepsis), and improved organ function [104,105].

## 8. Inhibition of TGF-β

Klotho blocks TGF-β action, as we have previously reviewed [16]. This is particularly important because overexpression of TGF-β contributes to aging in several ways. This cytokine, and other members of the TGF-β family, can promote cellular senescence, cell death, fibrosis, immune dysfunction, loss of muscle mass, as well as cancer progression [106,107,108,109]. Indeed, TGF-β is involved in a vast number of cellular responses. The TGF-β family includes 33 members. TGF-β is the best characterized, and it signals by a canonical pathway and numerous noncanonical pathways [110,111,112]. It is secreted in latent form, binds to connective tissue components, and is activated primarily by interaction with integrins, although other mechanisms apply. The three isoforms of TGF-β (TGF-β1 is the most abundant) all bind to the same membrane-bound signaling receptor. It consists of two chains; the type 1 chain is denoted TβRI (ALK5) and the type 2 chain TβRII. In the initial interaction, TGF-β binds to TβRII and TβRI to create a serine/threonine kinase complex. TβRII phosphorylates TβRI, which in the canonical pathway phosphorylates Smad2 and Smad3. These two Smads form a complex with Smad4 (the common Smad) that translocates into the nucleus. It then binds to DNA and regulates the transcription of numerous genes.

The TGF-β signaling receptor also activates key noncanonical pathways including ERK, JNK, p38 MAPK, PI3K/Akt, Rho-like GTPases and NF-κB [110,112]. Moreover, it can crosstalk with other pathways influencing a large proportion of cell functions, including other TGF-β family cytokines (BMPs, Nodal, myosin), Wnt, Notch, Hedgehog, Hippo (TAZ/YAP) and JAK/STAT. It can also crosstalk with vascular endothelial growth factor (VEGF), epidermal growth factor (EGF) and hepatocyte growth factor (HGF). This demonstrates the exceedingly high number of pathways that can be modified when increasing or decreasing TGF-β activity.

TGF-β is a key regulator of the immune system. It is secreted by regulatory T cells (Treg), macrophages and several other cell types. It exerts immunosuppressive or regulatory functions on dendritic cells, macrophages, B lymphocytes, effector T lymphocytes, NK cells and neutrophils [107,113]. As related to cancer, TGF-β acts as a tumor suppressor in the early stages of neoplasia, but at later stages it promotes cancer progression and metastasis [109].

Klotho inhibits TGF-β by binding to the signaling TGF-β receptor [39,41]. Indeed, it has been reported to attach to the TβRI and TβRII chains of the receptor. These inhibitory effects have been demonstrated in vivo, especially against fibrosis. For instance, Klotho or a short Klotho peptide (KL1 domain) prevented renal fibrosis in mice [114]. The inhibition of TGF-β family members by Klotho might be applicable, for example, to pulmonary fibrosis, chronic renal disease, diabetes, cirrhosis of the liver, systemic sclerosis and sarcopenia. However, applications are still at a preclinical stage.

## 9. Sarcopenia

Sarcopenia (loss of muscle mass and/or function) is a feature of old age, inflammation/inflammaging, several chronic diseases and cancer. Klotho deficient mice (*kl*/*kl* or knockout) have sarcopenia and, in humans, low Klotho levels associate with sarcopenia. This subject has been recently reviewed by others [42,115], and is only briefly covered in this manuscript. The pathogenesis of sarcopenia is complex and not fully understood. The underlying mechanisms likely differ considerably depending on the clinical context, such as aging, obesity, kidney disease, cancer and other conditions. Obesity (sarcopenic obesity) [115] and chronic kidney disease [116] are common factors that induce sarcopenia. Both are associated with decreased s-Klotho levels. Importantly, Klotho overexpression in mice (by viral gene transfer) ameliorated muscle mass and structure, although this appeared to be age dependent [45]. Interestingly, this treatment had an impact on the expression of all the groups of genes related to the hallmarks of aging [45].

The action of TGF-β and myostatin (another TGF-β family member) appears important, at least in some experimental models of sarcopenia. Myostatin reduces muscle mass, as occurs in senile muscle atrophy and cachexia [117]. Ohsawa et al. [41] reported that Klotho inhibits multiple members of the TGF-β family that can reduce muscle mass. This involved TGF-β1, myostatin, GDF11 and activins. This work was performed in vitro; however, the injection of a TGF-β inhibitory drug ameliorated muscle mass in Klotho knockout mice and old wild-type mice.

Other authors have reported that Klotho ameliorates muscle regeneration and healing in response to injury [118]. In renal failure, excess FGF23 and low Klotho induce muscle wasting [119]. It has been proposed that this is due to inflammation; particularly NF-κB activation and the release of inflammatory cytokines. Furthermore, high levels of FGF23 suppress the levels of Klotho and likely negate its activity.

The findings outlined above suggest that Klotho has potential for the treatment of sarcopenia. However, it is still unknown whether it can be applied to human sarcopenia treatment.

## 10. Antioxidant Functions

Klotho has major antioxidant properties, mainly by activating Nrf2 and FoxO. This promotes the expression of several antioxidant enzymes, including superoxide dismutase (SOD), glutathione (GSH)-associated enzymes, catalase, and the thioredoxin system [35,102,120]. Klotho activated Nrf2 in diseases of the kidneys, the brain and the cardiovascular system [37,121,122,123,124].

In the case of FOXO proteins, Klotho inhibits the IGF-1 receptor, which abrogates IGF-1 inhibition of FOXO. Then, the FOXO proteins migrate into the nucleus and mitigate oxidative stress through enzymes including catalase and manganese SOD [35]. For instance, Klotho protected rat hearts from ischemia–reperfusion injury (IRI) [38]. This depended on the inhibition of the IGF1R/PI3K/AKT pathway.

## 11. Protection Against Cellular Senescence

As mentioned previously, Klotho has been found to protect cells against senescence. This has been observed in kidney tubules [24], cardiovascular system [23,123], lungs [124], brain [25,125]; pancreatic β cells [26], and other sites.

Classically, cellular senescence was recognized as a feature of telomere attrition, especially in cells dividing in culture. However, cell senescence is also a response to cell injury, which can occur in many situations [126]. For example, it can be triggered by oxidative stress, mitochondrial dysfunction, DNA damage, ER stress, oncogene activation, cancer chemotherapy, inflammatory cytokines, and TGF-β. The two classical features are cell cycle arrest (G1/S or G2 phase), and the senescence-associated secretory phenotype (SASP) [127]. Senescence-associated β-galactosidase (SA-β-gal) is a marker that is frequently used to identify senescent cells. Activation of some pathways (especially p16 and p53-p21) leads to permanent cell-cycle arrest, although these cells can maintain some functions and have SASP. The SASP consists of the secretion of chemokines, inflammatory cytokines, growth factors and other mediators. However, the senescence markers and composition of the SASP can vary considerably from one cell type to another.

Cells that do not normally divide can nevertheless show other features of senescence, although this can be more difficult to establish. We examined senescence in pancreatic β cells [26]. Of interest, we observed that old mice had increased numbers of senescent β cells. Furthermore, the β cells of these old mice showed severe downregulation of Klotho expression. We examined β-cell senescence in vitro with the INS-1 pancreatic β cell line [26]. Senescence was induced by incubation with doxorubicin. The senescent cells displayed marked loss of Klotho expression. However, the addition Klotho to the cultures protected these cells against senescence. It improved mitochondrial function and reduced ROS production. Klotho also improved insulin secretion. These findings suggest that Klotho has a protective role in the endocrine pancreas, in accord with our previous studies [90,128].

## 12. Klotho Mitigates Vascular Aging

Pathology involving the arteries is a major negative aspect of aging. It is a frequent cause of death. Arterial calcification and atherosclerosis occur gradually with age. The occurrence of early (premature) vascular aging is precipitated by either smoking, diabetes, hypertension or chronic kidney disease. In cases of advanced renal failure, Klotho insufficiency leads to hyperphosphatemia and markedly increased FGF23 levels. These extremely high levels of FGF23 can activate FGFR4 in the absence of Klotho [129]. High FGF23 levels have multiple adverse effects such as endothelial-cell dysfunction, ventricular hypertrophy, heart failure and increased mortality [129]. In contrast to FGF23, high Klotho levels associate with reduced heart failure [48].

In Klotho-deficient mice, calcification of the arteries occurs rapidly and is a prominent feature [4]. In humans over 70 years old, calcium deposition in multiple vessels is common; notably in the aorta, peripheral arteries, coronary arteries and cerebral arteries [130]. In some cases, calcium accumulates mainly in the media of arteries (Monckeberg’s medial calcific sclerosis) [131]. However, calcium is often deposited in atherosclerotic plaques, including the most clinically relevant sites such as the coronary and cerebral arteries.

Atherosclerosis associates with hyperlipidemia, but inflammation is an important contributing factor [132]. Cholesterol crystals accumulate in the plaques, and these crystals are phagocytosed by macrophages. This results in the activation of the NLRP3 inflammasome. In this situation, thioredoxin-interacting protein (TXNIP), ROS and calcium deposits can probably all activate the inflammasome. Subsequent to this, plaque erosion or rupture leads to thrombosis and vessel occlusion [133].

Recently, in a clinical study, Hellou et al. [134] reported that circulating Klotho levels inversely correlated with calcification of the aorta and iliac arteries. Furthermore, low Klotho was independently associated with increased mortality. Interestingly, Klotho was identified in the wall of blood vessels, and circulating Klotho might not be the only factor. In several previous studies, others also reported that Klotho insufficiency associates with calcific vasculopathy and/or atherosclerosis [135,136,137,138]. Klotho appears to protect against vascular disease by reducing calcification, inflammation and endothelial cell injury.

## 13. Retinopathy

Diabetes promotes atherosclerotic disease in arteries of various sizes, but arterioles and capillaries can also be involved. Indeed, diabetic retinopathy (DR) is characterized by microangiopathy of the retina. In advanced DR cases (proliferative DR) there is angiogenesis, hemorrhage and fibrosis. This can progress to blindness. In the retina, Klotho is produced locally and appears essential to maintain normal function [139]. The findings suggest that Klotho mitigates DR by preventing epithelial–mesenchymal transition (EMT), ROS production, vascular endothelial growth factor (VEGF) release, and cell death by apoptosis [140,141]. To our knowledge, its use for clinical treatment has not been reported.

Klotho is also relevant to age-related macular degeneration (AMD). TGF-β2 (produced in the eye) is thought to be a major factor in the pathogenesis of AMD [142]. Increased vitreous TGF-β2 likely induces retinal fibrosis. In mice, intravitreal injection of Klotho protected against TGF-β2-induced EMT, and retinal epithelial-cell degeneration [142]. Klotho treatment ameliorated cell senescence and EMT. There was reduced cytosolic and mitochondrial oxidative stress. These reports in DR and AMD suggest that Klotho could useful in the treatment of some retinopathies.

## 14. Klotho and the Skin

Klotho is expressed in the skin and other epithelial tissues [2]. Strong positive staining was seen in the epidermis and skin appendages. The skin is a major target of aging, and subject to injury by ultraviolet (UV) radiation and many other factors. Cells may undergo senescence and apoptosis. It is a major target of inflammaging, as well as a wide variety of inflammatory diseases [143]. Despite this, there have been very few studies of Klotho function in the skin. Klotho protected human keratinocytes against UV radiation, possibly by inhibiting NF-κB [144]. It prevented endothelial-to-mesenchymal transition (EndMT) [145], and this also appeared to be related to diminished NF-κB activation. Recently, Humble et al. [146] examined the effect of a Klotho-containing serum on photoaging in a pilot study. The participants experienced improvement in photoaging, wrinkles and other changes. Larger studies are required to confirm these observations.

## 15. Tumor Suppressor Functions

Age is a major factor in the occurrence of cancer. Some hallmarks of aging are relevant to cancer—especially genomic instability (DNA damage), epigenetic alterations and chronic inflammation. However, cancer cells resist telomere attrition and cell cycle arrest (senescence).

Several studies have confirmed that Klotho exerts potent anti-tumor activity. This has been extensively reviewed by others [15,43,147,148,149], and is only briefly addressed here. Indeed, Klotho inhibits several pathways that promote cancer progression, including Wnt, IGF-1, TGF-β, NF-κB and others. Klotho expression is frequently suppressed in malignant tumors, and tumors expressing higher levels have a more favorable prognosis. Reduced Klotho expression is often related to increased promoter methylation. Klotho is protective in several types of cancer, such as breast, pancreas, colon and liver. Importantly, inducing the expression of Klotho, or treatment with s-Klotho, suppressed the growth of cancer cells, as reviewed [149]. Klotho suppressed cancer cell proliferation, invasion and colony formation. For instance, Abramovitz et al. [64] found that Klotho expression was low in pancreatic carcinoma. Klotho treatment, or overexpression of Klotho, reduced pancreatic cancer cell proliferation, both in vitro and in vivo. It countered IGF-1 and FGF pathways. Injection of the Klotho KL1 domain mimicked the anti-cancer activity of the larger s-Klotho (KL1/KL2). Overall, these studies highlight the potential of Klotho as an anti-cancer agent.

## 16. Klotho Against Neurodegenerative Diseases

The role of Klotho in the CNS is not precisely elucidated, but there is extensive evidence of neuroprotection. With age, Klotho levels decline in the CSF, and its expression is reduced in the brain. Generally, low levels of Klotho associate with decreased cognitive abilities. The protective function of the KL-VS haplotype in heterozygous form was discussed previously (Section 6). More direct evidence of Klotho protection has been observed in human cerebral organoids. Forced expression of Klotho by these cells decreased neuronal senescence [150]. Importantly, Klotho protected cultured cortical neurons against Aβ toxicity; reducing neuronal apoptosis and degeneration [151]. Interestingly, these investigators observed that Klotho is expressed mostly in the axons of cortical neurons [151]. This work establishes that Klotho has direct neuroprotective effects. In cultures of neural hippocampal precursor cells, others observed that Klotho augmented neuronal differentiation and decreased apoptosis [152]. From an in vivo point of view, Klotho protein injections in nonhuman primates improved their performance in cognitive tests [153].

Indeed, Klotho-dependent neuroprotection has been reported in many preclinical studies [154,155,156,157,158,159,160,161]. It exerts anti-inflammatory and neuroprotective effects [157]. It also ameliorates myelination in the CNS, which is particularly relevant to multiple sclerosis [154]. Recently, Roig-Soriano et al. [7] delivered secreted Klotho in mice with an adeno-associated viral (AAV9) vector. It was administered by combined i.v. and intra-cerebroventricular (ICV) injection. This resulted in increased systemic and cerebral Klotho production. The injected mice showed several benefits, including increased longevity, reduced muscle fibrosis, ameliorated muscle regeneration response and, in the brain, increased cellular markers of neurogenesis.

In Alzheimer’s disease, neuroinflammation and related inflammasome activation are likely key pathogenic factors [162,163,164,165,166,167]. Notably, Aβ and hyperphosphorylated Tau protein, which are major molecular targets, promote the activation the NLRP3 inflammasome [167,168]. Moreover, other molecular targets and other inflammasomes are most likely involved. Klotho reduces neuroinflammation. Notably, it suppresses ROS, TXNIP, NF-κB activation, NLRP3 inflammasome action, and neuronal cell death.

## 17. Therapy with Klotho and Klotho-Enhancing Agents

### 17.1. Preclinical Therapy with Proteins, Peptides and Gene Transfer

**Figure 3:**
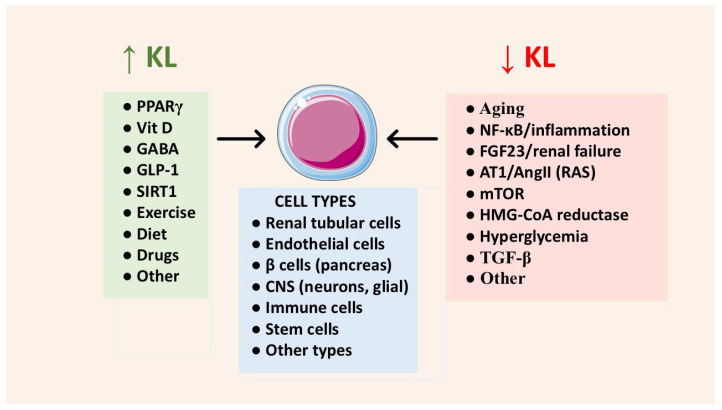
Several factors increase or decrease Klotho (KL) expression. This pertains to membrane-bound Klotho and/or s-Klotho, as reported in the text. **Abbreviations:** ↑, increased; ↓, decreased; Ang II, angiotensin II; AT1, angiotensin II receptor; FGF23, fibroblast growth factor 23; GABA, γ-aminobutyric acid; GLP-1, glucagon-like peptide 1; HMG-CoA reductase, the enzyme that is inhibited by statin drugs; KL, α-Klotho; mTOR, mechanistic target of rapamycin; NF-κB, nuclear factor κB; PPAR-γ, peroxisome proliferator-activated receptors γ; RAS, renin-angiotensin system; SIRT1, sirtuin 1; TGF-β, transforming growth factor β; Vit D, vitamin D.

The numerous antiaging functions of Klotho are summarized in Table 1. A surprising number of physiological mediators, drugs and other products alter Klotho expression [3,169] (Figure 3). With respect to aging, some downregulate Klotho and have a negative impact. This includes, for example, components of the renin-angiotensin system (RAS), NF-κB/inflammatory mediators, FGF23 (especially in renal disease), TGF-β, and hyperglycemia.

In preclinical work, the administration of either s-Klotho, the KL1 domain or Klotho-derived peptides has protected against renal, cardiovascular, metabolic and neurodegenerative diseases [3,5,7,12,141,170]. Anti-inflammatory, anti-fibrotic and neuroprotective effects were observed in several of these studies. Klotho therapy also exerted anti-cancer activities, as mentioned previously.

s-Klotho can bind to FGFR1 and act as coreceptor for FGF23. This requires the KL2 domain. The soluble KL1/KL2 form might be important to promote FGF23 action in blood vessels, but this remains to be confirmed. Of major interest, most (if not all) the blocking properties of s-Klotho can be ascribed to the KL1 domain. Thus, a KL1-derived peptide of 101 amino acids inhibited NF-κB [85,86]. A 30 amino-acid KL1 peptide blocked the TGF-β receptor [114]. Likewise, another 30 amino acid KL1 peptide (P6) attached to Wnt ligands and blocked their activity [40]. KL1 and these peptides are active in human cells, at least in vitro. However, to our knowledge, their therapeutic effectiveness in humans has not been determined.

### 17.2. Small Drugs That Increase Klotho Levels

Some clinical drugs, nutraceuticals and traditional medicines have been found to increase endogenous Klotho production, as reviewed [3,12,170]. This includes common drugs such as RAS inhibitors (losartan, valsartan), statins (atorvastatin, fluvastatin and others), mTOR inhibitors (rapamycin, everolimus), vitamin D and pentoxifylline. In mice, we found that γ-aminobutyric acid (GABA) systemic therapy increased Klotho in the kidneys, pancreatic β cells and serum [3,90]. GABA also increased Klotho expression in human pancreatic β cells [171,172].

Most types of clinical anti-diabetic drugs are effective at raising Klotho levels, such as metformin, glucagon-like peptide 1 (GLP-1) mimetics, peroxisome proliferator-activated receptor γ (PPAR-γ) agonists and sodium-glucose costransporter 2 (SGLT2) inhibitors [3,16]. This is possibly because hyperglycemia suppresses Klotho production. Well-known nutraceuticals (especially phytotherapeutics), such as astaxanthin, curcumin, ginseng and resveratrol also increase Klotho [3,141].

Drugs that cross the blood–brain barrier (BBB) are indicated for the treatment of central nervous system (CNS) conditions. Some senolytics (drugs that eliminate senescent cells) increased Klotho in the brain. Treatment with the senolytics dasatinib plus quercetin, which cross the BBB, increased Klotho in the kidney, whole brain, cerebellum and choroid plexus of old mice [173]. Urine Klotho was also increased. The application of telmisartan is an interesting possibility. It crosses the BBB, blocks the RAS (AT1 receptor antagonist), activates PPAR-γ and inhibits NF-κB [174]. Thus, it can stimulate Klotho expression by separate pathways. Telmisartan treatment appeared to augment Klotho in the brain of mice [175]. In APP/PS1 mutant mice it improved cognitive impairment, Aβ pathology and neuroinflammation [176].

The vast majority of these studies were performed in rodents. Often, only s-Klotho serum levels were examined. In general, Klotho was re-established to normal or near-normal levels in disease models where it had been diminished. Very little information is available on increasing Klotho levels in healthy animals. Because most of these drugs or substances exert multiple pharmacologic effects, it is unclear to what extent Klotho might contribute to disease amelioration.

### 17.3. Klotho Enhancement in Clinical Trials

Some evidence of drug-induced Klotho augmentation comes from clinical trials. Klotho increases of 5% to 25% have been reported. Drugs of several types have this property. For example, this was the case for renin-angiotensin system (RAS) inhibitors [177,178], a statin (fluvastatin) alone or combined with a RAS inhibitor (valsartan) [179], an mTOR inhibitor [180], anti-diabetic SGLT2 inhibitors [181], pentoxifylline [182], and vitamin D [183,184].

In a randomized controlled clinical trial, a dietary supplement (TRI 360^TM^) consisting of multiple vitamins (including vitamin D), minerals and ginseng powder boosted Klotho levels in subjects with psychological symptoms [185]. Klotho was prominently increased at days 90 and 180 of treatment, as compared to the placebo. This was associated with reduced oxidative stress and inflammatory biomarkers. Since multiple substances are in this supplement, it is unclear which enhanced Klotho; although both vitamin D and ginseng appear to have this property.

### 17.4. Role of Diet and Exercise

Multiple dietary factors have been reported to influence Klotho levels [186,187,188,189]. There have been relatively few clinical trials. Alterations in carbohydrates, fats, vitamins and minerals all appear to modify Klotho expression. Inflammatory dietary profiles inversely associate with serum Klotho levels [186]. Adherence to the Mediterranean Diet significantly increased Klotho [189]. However, in that study, only three items of the diet appeared to mediate most of the positive effect—fruits, dairy products and alcohol. Three other diets were compared and had no significant effect on Klotho. In a study of a similar population [187], the authors examined the contribution of many dietary components. After adjusting for age and sex, the only significant associations with higher Klotho levels that remained were more carbohydrate and total sugars, and less alcohol. A caveat in examining diet studies is the presence of numerous confounding factors. The results obtained in animal models are not necessarily applicable to humans. Klotho levels are highly variable in the human population, and sensitive to many factors. The levels reported with different Klotho assays can vary considerably [5,190].

The ability of exercise to increase Klotho has received considerable attention. This ties in with the well-known health benefits of physical activity and planned exercise. The relationship between exercise and Klotho has been examined by several investigators [191,192,193,194,195,196,197,198,199,200]. Various forms of exercise have been reported to increase Klotho, although there is not a general agreement on the best type, and/or the degree of Klotho enhancement. In a number of studies, the results were influenced by age, obesity, frailty, length of the exercise program and other factors. Of interest, Correa et al. [195] performed a meta analysis. They reviewed the findings from 12 reports involving 621 subjects aged 30 to 65 years. Klotho concentration increased significantly after chronic exercise training (minimum of 12 weeks). Interestingly, Klotho levels generally increased independently of the health condition or the exercise program, but with some exceptions. In conclusion, s-Klotho levels were generally increased after chronic exercise training. However, obesity appears to be a negative factor for Klotho enhancement [198,199,200]. The mechanisms by which exercise increases Klotho are not well understood, and this subject requires further investigation.

Some studies on diet, exercise, chronic diseases, aging and other aspects of Klotho biology are based on a single measurement of Klotho in each individual, and are not definitive. Furthermore, the specificity of some ELISA assays is not completely clear, making comparisons between studies difficult [5,10,190]. This has been a longstanding issue in Klotho research. An additional aspect to consider when analyzing Klotho effects is the concurrent FGF23 level (which is often not available). This is because high FGF23 suppresses the expression of Klotho, and is damaging to endothelial cells and the cardiovascular system [10,190].

## 18. Conclusions

The ability of Klotho to suppress aging has been known for almost thirty years. These antiaging properties are still not fully delineated, but cannot be ascribed to a single mechanism. Instead, we note several features that match the current hallmarks of aging, as reported in the literature. In mice, overexpression of Klotho modified the expression of all the groups of genes related to the hallmarks of aging. Furthermore, Klotho mitigates several major pathologies that are likely multifactorial and not easily explained by a single hallmark. Indeed, the antiaging properties of Klotho are exceptional. This is completely consistent with the fact that Klotho inhibits several pathways that have long been known to promote aging, such as TGF-β, IGF-1, NF-κB and Wnt.

In addition to this, the regulation of phosphate/calcium homeostasis is important. Klotho deficiency causes hyperphosphatemia, which is not one of the hallmarks but can mediate cellular injury. Other aspects such as protection against DNA damage, telomere attrition and stem cell depletion agree well with the hallmarks. However, in the case of Klotho, some of these aspects have not been examined extensively, and the actual mechanisms involved require more investigation. A major action of Klotho is the reduction in cellular senescence, which has been identified repeatedly. Another recurrent finding is the amelioration of mitochondrial function, and depletion of ROS. These are all important components of the aging hallmark classification.

Protection against inflammation might be the most important feature. Chronic inflammation is one of the major hallmarks of aging. Furthermore, inflammation exacerbates the other hallmarks. Examining the literature, it is evident that most of the diseases that are ameliorated by Klotho have a strong inflammatory component. In fact, Klotho can counteract both acute and chronic inflammation. This is consistent with Klotho’s ability to block NF-κB and the NLRP3 inflammasome. In addition, inflammation contributes to cell death by several pathways. In this regard, Klotho might also be effective against sepsis, and associated septic shock that has a high rate of fatal outcome.

Another key point of Klotho is its anti-fibrotic action. It has been shown to prevent fibrosis in major organs (kidneys, heart, lungs and liver), mainly through the inhibition of TGF-β and Wnt. At advanced stages fibrosis can lead to organ failure, and this is much more likely to occur in aged individuals. The blockade of TGF-β and myostatin also appear to protect against sarcopenia, at least in experimental models. Sarcopenia is a very common morbidity of aging, obesity, chronic diseases and cancer. The inhibition of TGF-β may also be relevant to the treatment of some retinopathies (e.g., diabetic, AMD). Finally, the antitumor functions are well documented, involve several mechanisms, and appear likely to be clinically applicable.

We examined several factors that increase or decrease Klotho. For example, hyperglycemia is a negative factor, and most anti-diabetic drugs increase Klotho. NF-κB (as in inflammation) and TGF-β (as in fibrosis) are other negative regulators of Klotho expression of clinical importance. They are potentially amenable to Klotho therapy. Practically all current drugs or other treatments that increase Klotho also have many other effects. In these cases, it can be extremely difficult to determine the contribution of Klotho. A major limitation remains the lack of clinical investigation of direct Klotho therapy, and this should be a prime goal of future research.

## Figures and Tables

**Table 1:** Summary of antiaging mechanisms of Klotho.

Mechanisms	Pathway/Target	Biological Effects	Relevance to Aging	References
Regulation of phosphate/calcium homeostasis	Phosphaturic effect; prevention of hyperphosphatemia and phosphatopathy	Reduced cellular senescence, oxidative stress, endothelial injury, TGF-β and other.	Delays vascular aging and tissue calcification.	[1,5,13,14,72,73,74]
Protection against DNA damage	DNA repair mechanisms	Reduced chromosomal DNA damage and mutations.	Delays cellular aging and reduces risk of cancer.	[20]
Protection against telomere attrition	Telomeres preserved by altering several telomerase regulatory components	Preservation of cellular replicative capacity.	Delays aging as related to cell senescence.	[21]
Reduction in cellular senescence	Multiple potential mechanisms, preservation of telomeres, reduced cell injury, blocked TGF-β	Preservation of cell replication and function in multiple tissues and organs.	Delayed aging and improved function of aging cells.	[23,24,25,26,123,124,125]
Preservation of mitochondrial function	Mechanisms not elucidated	Reduced reactive oxygen species (ROS).	Protection of cells against degraded mitochondrial function and oxidative stress.	[23,24,25,26,27]
Reduced inflammation and inflammaging	Inhibition of NF-κB and the NLRP3 inflammasome	Reduced chemokines, cytokines, and other inflammatory mediators; reduced cell injury and death.	Reduced inflammation mitigates all the hallmarks of aging.	[8,16,28,30,85,86,87,88,89,90,91,92,93,94,95,102,103,104,105]
Reduced endothelial cell injury	Multiple mechanisms (e.g., reduced cytokines, inflammasome activation, oxidative stress, phosphate injury)	Protection against vascular calcification, atherosclerosis and thrombosis.	Delays vascular aging, reduces risk of myocardial infarction and strokes.	[23,29,30]
Reduction in endoplasmic reticulum (ER) stress	Mechanism unknown	Improvement of proteostasis.	Reduction in defective and/or toxic protein accumulation as related to aging.	[31,32,33,34]
Improved resistance against ROS injury (oxidative stress)	Induction of Nrf2 and FOXOs; increased antioxidant enzymes	Reduction in oxidative cellular injury in many circumstances.	Improved cell survival and function.	[35,36,37,38]
Prevention of fibrosis	Blockage of TGF-β and Wnt	Reduced organ fibrosis, as related to inflammaging and other injury.	Improved organ function in aging.	[39,40,41,114]
Mitigation of sarcopenia	Reduced inflammation, blockade of TGF-β and myostatin	Improved muscle size and function.	Sarcopenia is a major negative factor of aging.	[41,42,45,115,116]
Tumor suppressor function	Inhibition of multiple pathways (TGF-β, IGF-1, NF-κB, Wnt and others)	Decreased carcinogenesis.	Decreased morbidity and mortality from cancer.	[15,43,147,148,149]
Delayed aging by biomarkers	Aging clocks addressing DNA methylation and several markers of aging	Improved health relative to chronological age.	Delayed aging by objective markers.	[44]
Reduced mortality	Multiple mechanisms	Low Klotho increases all-cause mortality, and risk of death in subjects with existing diseases.	Klotho improves lifespan.	[46,47,48,49,50,51]

## Data Availability

No new data were created or analyzed in this study.

## References

[1] Novillo-Sarmiento C., García-Sáez R.M., Rivas-Domínguez A., Torralba-Duque A., Rodelo-Haad C., Rodríguez-Ortiz M.E., Muñoz-Castañeda J.R., Pendón-RuizdeMier M.V. (2025). Phosphate and Inflammation in Health and Kidney Disease. Int. J. Mol. Sci..

[2] Lim K., Groen A., Molostvov G., Lu T., Lilley K.S., Snead D., James S., Wilkinson I.B., Ting S., Hsiao L.-L. (2015). alpha-Klotho Expression in Human Tissues. J. Clin. Endocrinol. Metab..

[3] Prud’homme G.J., Kurt M., Wang Q. (2022). Pathobiology of the Klotho Antiaging Protein and Therapeutic Considerations. Front. Aging.

[4] Kuro-O M., Matsumura Y., Aizawa H., Kawaguchi H., Suga T., Utsugi T., Ohyama Y., Kurabayashi M., Kaname T., Kume E. (1997). Mutation of the mouse klotho gene leads to a syndrome resembling ageing. Nature.

[5] Kuro-o M. (2019). The Klotho proteins in health and disease. Nat. Rev. Nephrol..

[6] Kurosu H., Yamamoto M., Clark J.D., Pastor J.V., Nandi A., Gurnani P., McGuinness O.P., Chikuda H., Yamaguchi M., Kawaguchi H. (2005). Suppression of aging in mice by the hormone Klotho. Science.

[7] Roig-Soriano J., Edo Á., Verdés S., Martín-Alonso C., Sánchez-De-Diego C., Rodriguez-Estevez L., Serrano A.L., Abraham C.R., Bosch A., Ventura F. (2025). Long-term effects of s-KL treatment in wild-type mice: Enhancing longevity, physical well-being, and neurological resilience. Mol. Ther..

[8] Lathe R., St. Clair D. (2023). Programmed ageing: Decline of stem cell renewal, immunosenescence, and Alzheimer’s disease. Biol. Rev. Camb. Philos. Soc..

[9] López-Otín C., Blasco M.A., Partridge L., Serrano M., Kroemer G. (2023). Hallmarks of aging: An expanding universe. Cell.

[10] Edmonston D., Grabner A., Wolf M. (2024). FGF23 and klotho at the intersection of kidney and cardiovascular disease. Nat. Rev. Cardiol..

[11] Hajare A.D., Dagar N., Gaikwad A.B. (2025). Klotho antiaging protein: Molecular mechanisms and therapeutic potential in diseases. Mol. Biomed..

[12] Kanbay M., Copur S., Ozbek L., Mutlu A., Cejka D., Ciceri P., Cozzolino M., Haarhaus M.L. (2023). Klotho: A potential therapeutic target in aging and neurodegeneration beyond chronic kidney disease—A comprehensive review from the ERA CKD-MBD working group. Clin. Kidney J..

[13] Kuro-o M. (2021). Aging and FGF23-klotho system. Vitam. Horm..

[14] Kuro-o M. (2021). Klotho and calciprotein particles as therapeutic targets against accelerated ageing. Clin. Sci..

[15] Ortega M.A., Boaru D.L., De Leon-Oliva D., De Castro-Martinez P., Minaya-Bravo A.M., Casanova-Martín C., Barrena-Blázquez S., Garcia-Montero C., Fraile-Martinez O., Lopez-Gonzalez L. (2025). The Impact of Klotho in Cancer: From Development and Progression to Therapeutic Potential. Genes.

[16] Prud’homme G.J., Wang Q. (2024). Anti-Inflammatory Role of the Klotho Protein and Relevance to Aging. Cells.

[17] Shen J., Bin W., Lin X., Lai Y., Lin X., Guan T., Liu H. (2025). Klotho Protein: A Multifaceted Guardian of Healthy Aging and Its Therapeutic Potential. Int. J. Nanomed..

[18] Bian A., Neyra J.A., Zhan M., Hu M.C. (2015). Klotho, stem cells, and aging. Clin. Interv. Aging.

[19] Dai D.F., Daneshgar N., Wang K., Liang P.I., Bosch D., Grueter C. (2025). Regulation of stem cell aging and cellular proliferation by Klotho-Sirt1 pathways in heart, kidney and small intestine. npj Aging.

[20] Nakayama S., Sun J., Horikoshi Y., Kamimura Y., Ike T., Fujino S., Kinugasa Y., Sasaki K., Nakashima A., Masaki T. (2023). Klotho protects chromosomal DNA from radiation-induced damage. J. Biochem..

[21] Ullah M., Sun Z. (2019). Klotho Deficiency Accelerates Stem Cells Aging by Impairing Telomerase Activity. J. Gerontol. A Biol. Sci. Med. Sci..

[22] Liu H., Fergusson M.M., Castilho R.M., Liu J., Cao L., Chen J., Malide D., Rovira I.I., Schimel D., Kuo C.J. (2007). Augmented Wnt signaling in a mammalian model of accelerated aging. Science.

[23] Carracedo J., Buendía P., Merino A., Madueño J.A., Peralbo E., Ortiz A., Martín-Malo A., Aljama P., Rodríguez M., Ramírez R. (2012). Klotho modulates the stress response in human senescent endothelial cells. Mech. Ageing Dev..

[24] Miao J., Huang J., Luo C., Ye H., Ling X., Wu Q., Shen W., Zhou L. (2021). Klotho retards renal fibrosis through targeting mitochondrial dysfunction and cellular senescence in renal tubular cells. Physiol. Rep..

[25] Roig-Soriano J., Griñán-Ferré C., Espinosa-Parrilla J.F., Abraham C.R., Bosch A., Pallàs M., Chillón M. (2022). AAV-mediated expression of secreted and transmembrane αKlotho isoforms rescues relevant aging hallmarks in senescent SAMP8 mice. Aging Cell.

[26] Wang Z., Ni Y., Lou Y.R., Prud’homme G.J., Wang Q. (2025). Klotho protects INS-1 pancreatic β-cells from senescence and enhances mitochondrial function. Front. Aging.

[27] Oskuye Z.Z., Mehri K., Khalilpour J., Nemati S., Hosseini L., Bafadam S., Abdollahzade N., Badalzadeh R. (2025). Klotho in age-related cardiovascular diseases: Insights into mitochondrial dysfunction and cell death. Int. J. Cardiol. Heart Vasc..

[28] Baechle J.J., Chen N., Makhijani P., Winer S., Furman D., Winer D.A. (2023). Chronic inflammation and the hallmarks of aging. Mol. Metab..

[29] Romero A., Dongil P., Valencia I., Vallejo S., Hipólito-Luengo Á.S., Díaz-Araya G., Bartha J.L., González-Arlanzón M.M., Rivilla F., de la Cuesta F. (2022). Pharmacological Blockade of NLRP3 Inflammasome/IL-1β-Positive Loop Mitigates Endothelial Cell Senescence and Dysfunction. Aging Dis..

[30] Yang K., Nie L., Huang Y., Zhang J., Xiao T., Guan X., Zhao J. (2012). Amelioration of uremic toxin indoxyl sulfate-induced endothelial cell dysfunction by Klotho protein. Toxicol. Lett..

[31] Banerjee S., Zhao Y., Sarkar P.S., Rosenblatt K.P., Tilton R.G., Choudhary S. (2013). Klotho ameliorates chemically induced endoplasmic reticulum (ER) stress signaling. Cell Physiol. Biochem..

[32] Song S., Gao P., Xiao H., Xu Y., Si L.Y. (2013). Klotho suppresses cardiomyocyte apoptosis in mice with stress-induced cardiac injury via downregulation of endoplasmic reticulum stress. PLoS ONE.

[33] Saleh R.O., Jabir M.S., Mohammed J.S., Ahmad I., Ganesan S., Shankhyan A., Nanda A., Ray S., Zwamel A.H., Hussein A.R. (2025). Exploring the Endoplasmic Reticulum’s Role in Alzheimer’s Disease and Its Potential as a Therapeutic Target. J. Biochem. Mol. Toxicol..

[34] Shah A., Karthikeyan T., Hashem S., Kumar R., Bhat A.A., Macha M.A. (2025). Protein misfolding and neurodegeneration: Mechanisms, implications, and therapeutic strategies. Adv. Protein. Chem. Struct. Biol..

[35] Donate-Correa J., Martín-Carro B., Cannata-Andía J.B., Mora-Fernández C., Navarro-González J.F. (2023). Klotho, Oxidative Stress, and Mitochondrial Damage in Kidney Disease. Antioxidants.

[36] Fu Y., Cao J., Wei X., Ge Y., Su Z., Yu D. (2023). Klotho alleviates contrast-induced acute kidney injury by suppressing oxidative stress, inflammation, and NF-KappaB/NLRP3-mediated pyroptosis. Int. Immunopharmacol..

[37] Maltese G., Psefteli P.M., Rizzo B., Srivastava S., Gnudi L., Mann G.E., Siow R.C. (2017). The anti-ageing hormone klotho induces Nrf2-mediated antioxidant defences in human aortic smooth muscle cells. J. Cell Mol. Med..

[38] Olejnik A., Radajewska A., Krzywonos-Zawadzka A., Bil-Lula I. (2023). Klotho inhibits IGF1R/PI3K/AKT signalling pathway and protects the heart from oxidative stress during ischemia/reperfusion injury. Sci. Rep..

[39] Doi S., Zou Y., Togao O., Pastor J.V., John G.B., Wang L., Shiizaki K., Gotschall R., Schiavi S., Yorioka N. (2011). Klotho inhibits transforming growth factor-beta1 (TGF-beta1) signaling and suppresses renal fibrosis and cancer metastasis in mice. J. Biol. Chem..

[40] Chen X., Tan H., Xu J., Tian Y., Yuan Q., Zuo Y., Chen Q., Hong X., Fu H., Hou F.F. (2022). Klotho-derived peptide 6 ameliorates diabetic kidney disease by targeting Wnt/β-catenin signaling. Kidney Int..

[41] Ohsawa Y., Ohtsubo H., Munekane A., Ohkubo K., Murakami T., Fujino M., Nishimatsu S.-I., Hagiwara H., Nishimura H., Kaneko R. (2023). Circulating α-Klotho Counteracts Transforming Growth Factor-β-Induced Sarcopenia. Am. J. Pathol..

[42] Sun C.C., Chen Y.J., Xiao J.L., Zhao Z., Tang C.F. (2025). The Emerging Role of alpha-Klotho as a Therapeutic Target for Sarcopenia: Underlying Mechanisms and Clinical Prospects. Compr. Physiol..

[43] Rubinek T., Wolf I. (2016). The Role of Alpha-Klotho as a Universal Tumor Suppressor. Vitam. Horm..

[44] Aczel D., Torma F., Jokai M., McGreevy K., Boros A., Seki Y. (2023). The Circulating Level of Klotho Is Not Dependent upon Physical Fitness and Age-Associated Methylation Increases at the Promoter Region of the Klotho Gene. Genes.

[45] Clemens Z., Sivakumar S., Pius A., Sahu A., Shinde S., Mamiya H., Luketich N., Cui J., Dixit P., Hoeck J.D. (2021). The biphasic and age-dependent impact of klotho on hallmarks of aging and skeletal muscle function. eLife.

[46] Charoenngam N., Ponvilawan B., Ungprasert P. (2020). Lower circulating soluble Klotho level is associated with increased risk of all-cause mortality in chronic kidney disease patients: A systematic review and meta-analysis. Int. Urol. Nephrol..

[47] Kresovich J.K., Bulka C.M. (2022). Low Serum Klotho Associated with All-cause Mortality Among a Nationally Representative Sample of American Adults. J. Gerontol. A Biol. Sci. Med. Sci..

[48] Mahenthiran A., Nunes S.A., Liu C.F., Leon S., Wilcox J., Tang W.H.W. (2026). Prognostic value of human serum alpha-klotho concentrations in patients with heart failure with reduced ejection fraction. Clin. Biochem..

[49] Sadr N., Avila C.J., Chung H., Siddiqui S., Basith A., Kassabo W., Qayyum R. (2024). The relationship between serum alpha-Klotho with all-cause and cause-specific mortality. Br. J. Clin. Pharmacol..

[50] Semba R.D., Cappola A.R., Sun K., Bandinelli S., Dalal M., Crasto C., Guralnik J.M., Ferrucci L. (2011). Plasma klotho and mortality risk in older community-dwelling adults. J. Gerontol. A Biol. Sci. Med. Sci..

[51] Wang J., Bai L., Ye Y., Chen X., Hu X., Peng Y. (2024). Sex differences in mortality risk and U-shaped relationship with klotho levels: A long-term cohort study. Exp. Gerontol..

[52] Abraham C.R., Li A. (2022). Aging-suppressor Klotho: Prospects in diagnostics and therapeutics. Ageing Res. Rev..

[53] Chen G., Liu Y., Goetz R., Fu L., Jayaraman S., Hu M.-C., Moe O.W., Liang G., Li X., Mohammadi M. (2018). α-Klotho is a non-enzymatic molecular scaffold for FGF23 hormone signalling. Nature.

[54] Erben R.G. (2018). Physiological Actions of Fibroblast Growth Factor-23. Front. Endocrinol..

[55] Neyra J.A., Hu M.C., Moe O.W. (2021). Klotho in Clinical Nephrology: Diagnostic and Therapeutic Implications. Clin. J. Am. Soc. Nephrol..

[56] Sun F., Liang P., Wang B., Liu W. (2023). The fibroblast growth factor-Klotho axis at molecular level. Open Life Sci..

[57] Suzuki Y., Kuzina E., An S.J., Tome F., Mohanty J., Li W., Lee S., Liu Y., Lax I., Schlessinger J. (2020). FGF23 contains two distinct high-affinity binding sites enabling bivalent interactions with α-Klotho. Proc. Natl. Acad. Sci. USA.

[58] Xu Y., Sun Z. (2015). Molecular basis of Klotho: From gene to function in aging. Endocr. Rev..

[59] Roig-Soriano J., Sánchez-De-Diego C., Esandi-Jauregui J., Verdés S., Abraham C.R., Bosch A., Ventura F., Chillón M. (2023). Differential toxicity profile of secreted and processed alpha-Klotho expression over mineral metabolism and bone microstructure. Sci. Rep..

[60] Dalton G.D., Xie J., An S.W., Huang C.L. (2017). New Insights into the Mechanism of Action of Soluble Klotho. Front. Endocrinol..

[61] Mencke R., Harms G., Moser J., van Meurs M., Diepstra A., Leuvenink H.G., Hillebrands J.L. (2017). Human alternative Klotho mRNA is a nonsense-mediated mRNA decay target inefficiently spliced in renal disease. JCI Insight.

[62] Urakawa I., Yamazaki Y., Shimada T., Iijima K., Hasegawa H., Okawa K., Fujita T., Fukumoto S., Yamashita T. (2006). Klotho converts canonical FGF receptor into a specific receptor for FGF23. Nature.

[63] Chen L., Fu L., Sun J., Huang Z., Fang M., Zinkle A., Liu X., Lu J., Pan Z., Wang Y. (2023). Structural basis for FGF hormone signalling. Nature.

[64] Abramovitz L., Rubinek T., Ligumsky H., Bose S., Barshack I., Avivi C., Kaufman B., Wolf I. (2011). KL1 internal repeat mediates klotho tumor suppressor activities and inhibits bFGF and IGF-I signaling in pancreatic cancer. Clin. Cancer Res..

[65] Smith E.R., Holt S.G., Hewitson T.D. (2019). αKlotho-FGF23 interactions and their role in kidney disease: A molecular insight. Cell. Mol. Life Sci..

[66] Xiao Z., King G., Mancarella S., Munkhsaikhan U., Cao L., Cai C., Quarles L.D. (2019). FGF23 expression is stimulated in transgenic alpha-Klotho longevity mouse model. JCI Insight.

[67] Chen T.H., Kuro-o M., Chen C.H., Sue Y.M., Chen Y.C., Wu H.H., Cheng C.Y. (2013). The secreted Klotho protein restores phosphate retention and suppresses accelerated aging in Klotho mutant mice. Eur. J. Pharmacol..

[68] Agrawal A., Ni P., Agoro R., White K.E., DiMarchi R.D. (2021). Identification of a second Klotho interaction site in the C terminus of FGF23. Cell Rep..

[69] Saar-Kovrov V., Donners M.M.P.C., van der Vorst E.P.C. (2021). Shedding of Klotho: Functional Implications in Chronic Kidney Disease and Associated Vascular Disease. Front. Cardiovasc. Med..

[70] Zou D., Wu W., He Y., Ma S., Gao J. (2018). The role of klotho in chronic kidney disease. BMC Nephrol..

[71] Latic N., Erben R.G. (2021). FGF23 and Vitamin D Metabolism. JBMR Plus.

[72] Ito N., Fukumoto S. (2021). Congenital Hyperphosphatemic Conditions Caused by the Deficient Activity of FGF23. Calcif. Tissue Int..

[73] Rausch S., Föller M. (2022). The regulation of FGF23 under physiological and pathophysiological conditions. Pflugers Arch..

[74] Ichikawa S., Imel E.A., Kreiter M.L., Yu X., Mackenzie D.S., Sorenson A.H., Goetz R., Mohammadi M., White K.E., Econs M.J. (2007). A homozygous missense mutation in human KLOTHO causes severe tumoral calcinosis. J. Clin. Investig..

[75] Chong W.H., Molinolo A.A., Chen C.C., Collins M.T. (2011). Tumor-induced osteomalacia. Endocr. Relat. Cancer.

[76] Brownstein C.A., Adler F., Nelson-Williams C., Iijima J., Li P., Imura A., Nabeshima Y.-I., Reyes-Mugica M., Carpenter T.O., Lifton R.P. (2008). A translocation causing increased alpha-klotho level results in hypophosphatemic rickets and hyperparathyroidism. Proc. Natl. Acad. Sci. USA.

[77] Mengel-From J., Soerensen M., Nygaard M., McGue M., Christensen K., Christiansen L.J. (2016). Genetic Variants in KLOTHO Associate with Cognitive Function in the Oldest Old Group. Gerontol. A Biol. Sci. Med. Sci..

[78] Di Bona D., Accardi G., Virruso C., Candore G., Caruso C. (2014). Association of Klotho polymorphisms with healthy aging: A systematic review and meta-analysis. Rejuvenation Res..

[79] Driscoll I.F., Lose S., Ma Y., Bendlin B.B., Gallagher C., Johnson S.C., Asthana S., Hermann B., Sager M.A., Blennow K. (2024). KLOTHO KL-VS heterozygosity is associated with diminished age-related neuroinflammation, neurodegeneration, and synaptic dysfunction in older cognitively unimpaired adults. Alzheimer’s Dement..

[80] Cook N., Driscoll I., Gaitán J.M., Glittenberg M., Betthauser T.J., Carlsson C.M., Johnson S.C., Asthana S., Zetterberg H., Blennow K. (2024). Amyloid-beta positivity is less prevalent in cognitively unimpaired KLOTHO KL-VS heterozygotes. J. Alzheimer’s Dis..

[81] Arking D.E., Atzmon G., Arking A., Barzilai N., Dietz H.C. (2005). Association between a functional variant of the KLOTHO gene and high-density lipoprotein cholesterol, blood pressure, stroke, and longevity. Circ. Res..

[82] Mendoza-Carrera F., Farías-Basulto A., Gómez-García E.F., Torre L.d.C.R.d.l., Cueto-Manzano A.M., Cortés-Sanabria L., Pérez-Coria M., Vázquez-Rivera G.E. (2024). Association of KLOTHO gene variants with metabolic and renal function parameters in Mexican patients living with type 2 diabetes. J. Diabetes Metab. Disord..

[83] Mussbacher M., Salzmann M., Brostjan C., Hoesel B., Schoergenhofer C., Datler H., Hohensinner P., Basílio J., Petzelbauer P., Assinger A. (2019). Cell Type-Specific Roles of NF-κB Linking Inflammation and Thrombosis. Front. Immunol..

[84] Mussbacher M., Derler M., Basílio J., Schmid J.A. (2023). NF-κB in monocytes and macrophages—An inflammatory master regulator in multitalented immune cells. Front. Immunol..

[85] Buendía P., Carracedo J., Soriano S., Madueño J.A., Ortiz A., Martín-Malo A., Aljama P., Ramírez R. (2015). α-Klotho Prevents NFκB Translocation and Protects Endothelial Cell from Senescence Induced by Uremia. J. Gerontol. A Biol. Sci. Med. Sci..

[86] Buendía P., Ramírez R., Aljama P., Carracedo J. (2016). α-Klotho Prevents Translocation of NFκB. Vitam. Horm..

[87] Guo Y., Zhuang X., Huang Z., Zou J., Yang D., Hu X., Du Z., Wang L., Liao X. (2018). Klotho protects the heart from hyperglycemia-induced injury by inactivating ROS and NF-kappaB-mediated inflammation both in vitro and in vivo. Biochim. Biophys. Acta Mol. Basis Dis..

[88] Li L., Wang Y., Gao W., Yuan C., Zhang S., Zhou H., Huang M., Yao X. (2015). Klotho Reduction in Alveolar Macrophages Contributes to Cigarette Smoke Extract-induced Inflammation in Chronic Obstructive Pulmonary Disease. J. Biol. Chem..

[89] M Maekawa Y., Ishikawa K., Yasuda O., Oguro R., Hanasaki H., Kida I., Takemura Y., Ohishi M., Katsuya T., Rakugi H. (2009). Klotho suppresses TNF-alpha-induced expression of adhesion molecules in the endothelium and attenuates NF-kappaB activation. Endocrine.

[90] Prud’homme G.J., Glinka Y., Kurt M., Liu W., Wang Q. (2017). The antiaging protein Klotho is induced by GABA therapy and exerts protective and stimulatory effects on pancreatic β cells. Biochem. Biophys. Res. Commun..

[91] Tsai K.-D., Lee Y.-C., Chen B.-Y., Wu L.-S., Liang S.-Y., Liu M.-Y., Hung Y.-W., Hsu H.-L., Chen P.-Q., Shieh J.-C. (2023). Recombinant Klotho attenuates IFNγ receptor signaling and SAMHD1 expression through blocking NF-κB translocation in glomerular mesangial cells. Int. J. Med. Sci..

[92] Wang Y., Wang K., Bao Y., Zhang T., Ainiwaer D., Xiong X., Wang G., Sun Z. (2022). The serum soluble Klotho alleviates cardiac aging and regulates M2a/M2c macrophage polarization via inhibiting TLR4/Myd88/NF-κB pathway. Tissue Cell.

[93] Typiak M., Piwkowska A. (2021). Antiinflammatory Actions of Klotho: Implications for Therapy of Diabetic Nephropathy. Int. J. Mol. Sci..

[94] Yu S., Yang H., Guo X., Sun Y. (2021). Klotho attenuates angiotensin II-induced cardiotoxicity through suppression of necroptosis and oxidative stress. Mol. Med. Rep..

[95] Zhao Y., Banerjee S., Dey N., LeJeune W.S., Sarkar P.S., Brobey R., Rosenblatt K.P., Tilton R.G., Choudhary S. (2011). Klotho depletion contributes to increased inflammation in kidney of the db/db mouse model of diabetes via RelA (serine)536 phosphorylation. Diabetes.

[96] Jin X., Ma Y., Liu D., Huang Y. (2023). Role of pyroptosis in the pathogenesis and treatment of diseases. MedComm.

[97] Qian X., Sun J., Li F., Xu L., Hu X., Dong N., Li G. (2025). Inflammasomes as the molecular hub of cardiovascular-metabolic-immune comorbidity networks. Front. Immunol..

[98] Paerewijck O., Lamkanfi M. (2022). The human inflammasomes. Mol. Aspects Med..

[99] Fu J., Wu H. (2023). Structural Mechanisms of NLRP3 Inflammasome Assembly and Activation. Annu. Rev. Immunol..

[100] Swanson K.V., Deng M., Ting J.P. (2019). The NLRP3 inflammasome: Molecular activation and regulation to therapeutics. Nat. Rev. Immunol..

[101] Vande Walle L., Lamkanfi M. (2023). Drugging the NLRP3 inflammasome: From signalling mechanisms to therapeutic targets. Nat. Rev. Drug Discov..

[102] Wei Z., Liu J., Liu H., Zhang X. (2025). Klotho alleviates sepsis-associated myocardial inflammation and apoptosis. Eur. J. Pharmacol..

[103] Al-Kadi A., Anter A., Rofaeil R.R., Sayed-Ahmed M.M., Ahmed A.F. (2025). Klotho: A multifaceted protector in sepsis-induced organ damage and a potential therapeutic target. World J. Crit. Care Med..

[104] Jou-Valencia D.B., Molema G., Popa E., Aslan A., van Dijk F.M., Mencke R., Hillebrands J.-L., Heeringa P., Hoenderop J.G., Zijlstra J.G. (2018). Renal Klotho is Reduced in Septic Patients and Pretreatment with Recombinant Klotho Attenuates Organ Injury in Lipopolysaccharide-Challenged Mice. Crit. Care Med..

[105] Li X., Zhai Y., Yao Q., The E., Ao L., Fullerton D.A., Yu K.-J., Meng X. (2025). Aging Impairs the Capacity of Cardiac Functional Recovery Following Endotoxemia: Modulation of Myocardial Klotho Level for Remedy. J. Surg. Res..

[106] Bhardwaj S., Gautam R.K., Kushwaha S. (2025). From senescence to scarring: Exploring TGF-beta signaling in cellular aging, fibrotic remodeling, and pulmonary fibrosis. Cytokine Growth Factor Rev..

[107] Prud’homme G.J. (2007). Pathobiology of transforming growth factor beta in cancer, fibrosis and immunologic disease, and therapeutic considerations. Lab. Investig..

[108] Ren L.L., Miao H., Wang Y.N., Liu F., Li P., Zhao Y.Y. (2023). TGF-β as A Master Regulator of Aging-Associated Tissue Fibrosis. Aging Dis..

[109] Zhao H., Yang F., Yang J., Yang S. (2026). SMAD signaling in cancer: Integrative roles in tumor progression, immune evasion, and therapeutic resistance. Cytokine.

[110] Mullen A.C., Wrana J.L. (2017). TGF-beta Family Signaling in Embryonic and Somatic Stem-Cell Renewal and Differentiation. Cold Spring Harb. Perspect. Biol..

[111] Derynck R., Budi E.H. (2019). Specificity, versatility, and control of TGF-β family signaling. Sci. Signal..

[112] Liao J., Wu T., Zhang Q., Shen P., Huang Z., Wang J., Zhang P., Lin S., Pi J., Zhang N. (2026). TGF-beta/BMP signaling in skeletal biology: Molecular mechanisms, regulatory networks, and therapeutic implications in development, regeneration, and disease. Bone Res..

[113] de Streel G., Lucas S. (2021). Targeting immunosuppression by TGF-beta1 for cancer immunotherapy. Biochem. Pharmacol..

[114] Yuan Q., Ren Q., Li L., Tan H., Lu M., Tian Y., Huang L., Zhao B., Fu H., Hou F.F. (2022). A Klotho-derived peptide protects against kidney fibrosis by targeting TGF-beta signaling. Nat. Commun..

[115] Ariadel-Cobo D.G., Estébanez B., González-Arnáiz E., García-Pérez M.P., Rivera-Viloria M., de la Maza B.P., Barajas-Galindo D.E., García-Sastre D., Ballesteros-Pomar M.D., Cuevas M.J. (2025). Influence of Klotho Protein Levels in Obesity and Sarcopenia: A Systematic Review. Int. J. Mol. Sci..

[116] Chang J., Liang Y., Sun P., Fang X., Sun Q. (2025). Molecular and Cellular Mechanisms Linking Chronic Kidney Disease and Sarcopenia in Aging: An Integrated Perspective. Clin. Interv. Aging.

[117] Lee S.J. (2023). Myostatin: A Skeletal Muscle Chalone. Annu. Rev. Physiol..

[118] Sahu A., Mamiya H., Shinde S.N., Cheikhi A., Winter L.L., Vo N.V., Stolz D., Roginskaya V., Tang W.-Y., St. Croix C. (2018). Age-related declines in α-Klotho drive progenitor cell mitochondrial dysfunction and impaired muscle regeneration. Nat. Commun..

[119] Elsurer Afsar R., Afsar B., Ikizler T.A. (2023). Fibroblast Growth Factor 23 and Muscle Wasting: A Metabolic Point of View. Kidney Int. Rep..

[120] Zeng Y., Xu G., Feng C., Cai D., Wu S., Liu Y., Chen Y., Ma W. (2023). Klotho inhibits the activation of the NLRP3 inflammasome to alleviate lipopolysaccharide-induced inflammatory injury in a549 cells and restore mitochondrial function through SIRT1/Nrf2 signaling pathway. Chin. J. Physiol..

[121] Xiang T., Luo X., Ye L., Huang H., Wu Y. (2022). Klotho alleviates NLRP3 inflammasome-mediated neuroinflammation in a temporal lobe epilepsy rat model by activating the Nrf2 signaling pathway. Epilepsy Behav..

[122] Xing L., Guo H., Meng S., Zhu B., Fang J., Huang J., Chen J., Wang Y., Wang L., Yao X. (2021). Klotho ameliorates diabetic nephropathy by activating Nrf2 signaling pathway in podocytes. Biochem. Biophys. Res. Commun..

[123] Xu Y., You J., Yao J., Hou B., Wang W., Hao Z. (2025). Klotho alleviates oxidative stress and mitochondrial dysfunction through the Nrf2/HO-1 pathway, thereby reducing renal senescence induced by calcium oxalate crystals. Urolithiasis.

[124] Li M., Chen B., Sun S., Wang K., Wang Y., Wu J. (2025). Klotho Regulates Club Cell Senescence and Differentiation in Chronic Obstructive Pulmonary Disease. Cell Prolif..

[125] Martínez-Cué C., Rueda N. (2020). Cellular Senescence in Neurodegenerative Diseases. Front. Cell Neurosci..

[126] Braga C.E.H.P., de Brito J.S., Ribeiro M., Coutinho-Wolino K.S., Regis B., Calixto B., Rodrigues R.C.B., Wang A.Y.-M., Stenvinkel P., Mafra D. (2026). Premature aging in chronic kidney disease: Decoding senescence biomarkers and therapeutic opportunities. Biochimie.

[127] Sun L., Chen C. (2025). Senescence in Aging and Alzheimer’s Disease. Aging Dis..

[128] Prud’homme G.J., Glinka Y., Kurt M., Liu W., Wang Q. (2020). Systemic Klotho therapy protects against insulitis and enhances beta-cell mass in NOD mice. Biochem. Biophys. Res. Commun..

[129] Diaz-Haaz D.I., Espinoza-Pérez E.A., Aguilar-Alonso J.A., Megchún-Hernández M., Gonzalez-Diaz F., García-Chong N.R. (2025). Effects of Fibroblast Growth Factor 23 (FGF23) on the Cardiovascular System: A Review of Literature. Cureus.

[130] Rocha-Singh K.J., Zeller T., Jaff M.R. (2014). Peripheral arterial calcification: Prevalence, mechanism, detection, and clinical implications. Catheter. Cardiovasc. Interv..

[131] Doherty T.M., Fitzpatrick L.A., Inoue D., Qiao J.-H., Fishbein M.C., Detrano R.C., Shah P.K., Rajavashisth T.B. (2004). Molecular, endocrine, and genetic mechanisms of arterial calcification. Endocr. Rev..

[132] Barbu E., Popescu M.R., Popescu A.C., Balanescu S.M. (2022). Inflammation as A Precursor of Atherothrombosis, Diabetes and Early Vascular Aging. Int. J. Mol. Sci..

[133] Yan A., Gotlieb A.I. (2023). The microenvironment of the atheroma expresses phenotypes of plaque instability. Cardiovasc. Pathol..

[134] Hellou E., Kalhor P., Babaie A., Akhiyat N., Hamidabad N.M., Nardi V., Manzato M., Nogami K., Copley E., O Lerman L. (2025). Reduced Circulating α-Klotho Levels Are Associated with Elevated Mortality and Arterial Calcifications of Aorta and Iliac Arteries. J. Am. Heart Assoc..

[135] Mencke R., Hillebrands J.L., on behalf of NIGRAM consortium (2017). The role of the anti-ageing protein Klotho in vascular physiology and pathophysiology. Ageing Res. Rev..

[136] Olejnik A., Franczak A., Krzywonos-Zawadzka A., Kałużna-Oleksy M., Bil-Lula I. (2018). The Biological Role of Klotho Protein in the Development of Cardiovascular Diseases. BioMed Res. Int..

[137] Tyurenkov I.N., Perfilova V.N., Nesterova A.A., Glinka Y. (2021). Klotho Protein and Cardio-Vascular System. Biochemistry.

[138] Tanriover C., Copur S., Mutlu A., Peltek I.B., Galassi A., Ciceri P., Cozzolino M., Kanbay M. (2023). Early aging and premature vascular aging in chronic kidney disease. Clin. Kidney J..

[139] Reish N.J., Maltare A., McKeown A.S., Laszczyk A.M., Kraft T.W., Gross A.K., King G.D. (2023). The age-regulating protein klotho is vital to sustain retinal function. Investig. Ophthalmol. Vis. Sci..

[140] Puddu A., Maggi D.C. (2023). Klotho: A new therapeutic target in diabetic retinopathy?. World J. Diabetes.

[141] Tang A., Zhang Y., Wu L., Lin Y., Lv L., Zhao L., Xu B., Huang Y., Li M. (2023). Klotho’s impact on diabetic nephropathy and its emerging connection to diabetic retinopathy. Front. Endocrinol..

[142] Jang H.Y., Kim S.J., Park K.S., Kim J.H. (2023). Klotho prevents transforming growth factor-β2-induced senescent-like morphological changes in the retinal pigment epithelium. Cell Death Dis..

[143] Andrzejczak K., Sternak A., Witkowski W., Ponikowska M. (2025). Inflammation-Driven Molecular Ageing in Chronic Inflammatory Skin Diseases: Is There a Role for Biologic Therapies?. Cells.

[144] Zhang B., Xu J., Quan Z., Qian M., Liu W., Zheng W., Yin F., Du J., Zhi Y., Song N. (2018). Klotho Protein Protects Human Keratinocytes from UVB-Induced Damage Possibly by Reducing Expression and Nuclear Translocation of NF-κB. Med. Sci. Monit..

[145] Romano E., Rosa I., Sgambati E., Fioretto B.S., Manetti M. (2025). Soluble alpha-Klotho protects dermal microvascular endothelial cells against endothelial-to-mesenchymal transition. Tissue Cell.

[146] Humble G., Dias S., Quintana S., Tatarinova T.V., Grimes P. (2025). A Pilot Study Evaluating Efficacy of a Topical Klotho Antiaging Serum to Treatable Visible Photoaging. J. Drugs Dermatol..

[147] Ligumsky H., Merenbakh-Lamin K., Keren-Khadmy N., Wolf I., Rubinek T. (2022). The role of α-klotho in human cancer: Molecular and clinical aspects. Oncogene.

[148] Mota J., Lima A.M.M., Gomes J.I.S., de Andrade M.S., Brito H.O., Silva M.M.A.L., Faustino-Rocha A.I., Oliveira P.A., Lopes F.F., Gil da Costa R.M. (2023). Klotho in Cancer: Potential Diagnostic and Prognostic Applications. Diagnostics.

[149] Sachdeva A., Gouge J., Kontovounisios C., Nikolaou S., Ashworth A., Lim K., Chong I. (2020). Klotho and the Treatment of Human Malignancies. Cancers.

[150] Shaker M.R., Aguado J., Chaggar H.K., Wolvetang E.J. (2021). Klotho inhibits neuronal senescence in human brain organoids. npj Aging Mech. Dis..

[151] Shaker M.R., Salloum-Asfar S., Taha R.Z., Javed I., Wolvetang E.J. (2025). Klotho overexpression protects human cortical neurons from beta-amyloid induced neuronal toxicity. Mol. Brain.

[152] Dias G.P., Murphy T., Stangl D., Ahmet S., Morisse B., Nix A., Aimone L.J., Aimone J.B., Kuro-O M., Gage F.H. (2021). Intermittent fasting enhances long-term memory consolidation, adult hippocampal neurogenesis, and expression of longevity gene Klotho. Mol. Psychiatry.

[153] Castner S.A., Gupta S., Wang D., Moreno A.J., Park C., Chen C., Poon Y., Groen A., Greenberg K., David N. (2023). Longevity factor klotho enhances cognition in aged nonhuman primates. Nat. Aging.

[154] Chen C.-D., Sloane J.A., Li H., Aytan N., Giannaris E.L., Zeldich E., Hinman J.D., Dedeoglu A., Rosene D.L., Bansal R. (2013). The antiaging protein Klotho enhances oligodendrocyte maturation and myelination of the CNS. J. Neurosci..

[155] Dubal D.B., Zhu L., Sanchez P.E., Worden K., Broestl L., Johnson E., Ho K., Yu G.-Q., Kim D., Betourne A. (2015). Life extension factor klotho prevents mortality and enhances cognition in hAPP transgenic mice. J. Neurosci..

[156] Gupta S., Moreno A.J., Wang D., Leon J., Chen C., Hahn O., Poon Y., Greenberg K., David N., Wyss-Coray T. (2022). vKL1 domain of longevity factor klotho mimics the metabolome of cognitive stimulation and enhances cognition in young and aging mice. J. Neurosci..

[157] López-Valdés H.E., Hernández-Lucas M., Rodríguez-Fabián G.D.J., Esteban-Román N.F., Gutiérrez-Juárez R., Arrieta-Cruz I., Martínez-Coria H. (2025). The Anti-Inflammatory Actions of Soluble Klotho in Brain Aging and Its Main Associated Diseases. Int. J. Mol. Sci..

[158] Nagai R., Saito Y., Ohyama Y., Aizawa H., Suga T., Nakamura T., Kurabayashi M., Kuro-O M. (2000). Endothelial dysfunction in the klotho mouse and downregulation of klotho gene expression in various animal models of vascular and metabolic diseases. Cell Mol. Life Sci..

[159] Orellana A.M., Mazucanti C.H., Dos Anjos L.P., de Sá Lima L., Kawamoto E.M., Scavone C. (2023). Klotho increases antioxidant defenses in astrocytes and ubiquitin-proteasome activity in neurons. Sci. Rep..

[160] Zhao Y., Zeng C.Y., Li X.H., Yang T.T., Kuang X., Du J.R. (2020). Klotho overexpression improves amyloid-β clearance and cognition in the APP/PS1 mouse model of Alzheimer’s disease. Aging Cell.

[161] Zhu L., Stein L.R., Kim D., Ho K., Yu G.-Q., Zhan L., Larsson T.E., Mucke L. (2018). Klotho controls the brain-immune system interface in the choroid plexus. Proc. Natl. Acad. Sci. USA.

[162] Han Y.H., Liu X.D., Jin M.H., Sun H.N., Kwon T. (2023). Role of NLRP3 inflammasome-mediated neuronal pyroptosis and neuroinflammation in neurodegenerative diseases. Inflamm. Res..

[163] Huang Y., Li X., Luo G., Wang J., Li R., Zhou C., Wan T., Yang F. (2022). Pyroptosis as a candidate therapeutic target for Alzheimer’s disease. Front. Aging Neurosci..

[164] Jose S., Groves N.J., Roper K.E., Gordon R. (2022). Mechanisms of NLRP3 activation and pathology during neurodegeneration. Int. J. Biochem. Cell Biol..

[165] Moonen S., Koper M.J., Van Schoor E., Schaeverbeke J.M., Vandenberghe R., von Arnim C.A.F., Tousseyn T., De Strooper B., Thal D.R. (2023). Pyroptosis in Alzheimer’s disease: Cell type-specific activation in microglia, astrocytes and neurons. Acta Neuropathol..

[166] Singh J., Habean M.L., Panicker N. (2023). Inflammasome assembly in neurodegenerative diseases. Trends Neurosci..

[167] McGroarty J., Salinas S., Evans H., Jimenez B., Tran V., Kadavakollu S., Vashist A., Atluri V. (2025). Inflammasome-Mediated Neuroinflammation: A Key Driver in Alzheimer’s Disease Pathogenesis. Biomolecules.

[168] Li Z., Gong C. (2025). NLRP3 inflammasome in Alzheimer’s disease: Molecular mechanisms and emerging therapies. Front. Immunol..

[169] Vogt J., Föller M. (2025). Regulation of αKlotho. Cell Physiol. Biochem..

[170] Poursistany H., Azar S.T., Azar M.T., Raeisi S. (2023). The current and emerging Klotho-enhancement strategies. Biochem. Biophys. Res. Commun..

[171] Liu W., Lau H.K., Son D.O., Jin T., Yang Y., Zhang Z., Li Y., Prud’homme G.J., Wang Q. (2021). Combined use of GABA and sitagliptin promotes human beta-cell proliferation and reduces apoptosis. J. Endocrinol..

[172] Son D.O., Liu W., Li X., Prud’homme G.J., Wang Q. (2019). Combined effect of GABA and glucagon-like peptide-1 receptor agonist on cytokine-induced apoptosis in pancreatic β-cell line and isolated human islets. J. Diabetes.

[173] Zhu Y., Prata L.G.L., Gerdes E.O.W., Netto J.M.E., Pirtskhalava T., Giorgadze N., Tripathi U., Inman C.L., Johnson K.O., Xue A. (2022). Orally-active, clinically-translatable senolytics restore α-Klotho in mice and humans. eBioMedicine.

[174] Quan W., Xu C.-S., Li X.-C., Yang C., Lan T., Wang M.-Y., Yu D.-H., Tang F., Wang Z.-F., Li Z.-Q. (2023). Telmisartan inhibits microglia-induced neurotoxic A1 astrocyte conversion via PPARγ-mediated NF-κB/p65 degradation. Int. Immunopharmacol..

[175] Yu F.Q., Peng H., Xing H.Y., Li G., Li M. (2011). Effect and mechanisms of Telmisartan on the expression of klotho protein in the brain of aging mice. J. Brain Nerv. Dis..

[176] Fu X.-X., Wei B., Cao H.-M., Duan R., Deng Y., Lian H.-W., Zhang Y.-D., Jiang T. (2023). Telmisartan Alleviates Alzheimer’s Disease-Related Neuropathologies and Cognitive Impairments. J. Alzheimer’s Dis..

[177] Karalliedde J., Maltese G., Hill B., Viberti G., Gnudi L. (2013). Effect of renin-angiotensin system blockade on soluble Klotho in patients with type 2 diabetes, systolic hypertension, and albuminuria. Clin. J. Am. Soc. Nephrol..

[178] Lim S.C., Liu J.J., Subramaniam T., Sum C.F. (2014). Elevated circulating alpha-klotho by angiotensin II receptor blocker losartan is associated with reduction of albuminuria in type 2 diabetic patients. J. Renin Angiotensin Aldosterone Syst..

[179] Janić M., Lunder M., Novaković S., Škerl P., Šabovič M. (2019). Expression of Longevity Genes Induced by a Low-Dose Fluvastatin and Valsartan Combination with the Potential to Prevent/Treat “Aging-Related Disorders”. Int. J. Mol. Sci..

[180] Mizusaki K., Hasuike Y., Kimura T., Nagasawa Y., Kuragano T., Yamada Y., Nojima M., Yamamoto S., Nakanishi T., Ishihara M. (2019). Inhibition of the Mammalian Target of Rapamycin May Augment the Increase in Soluble Klotho Levels in Renal Transplantation Recipients. Blood Purif..

[181] Mora-Fernández C., Sánchez-Niño M.D., Donate-Correa J., Martín-Núñez E., Pérez-Delgado N., Valiño-Rivas L., Fernández-Fernández B., Ortiz A., Navarro-González J.F. (2022). Sodium-glucose co-transporter-2 inhibitors increase Klotho in patients with diabetic kidney disease: A clinical and experimental study. Biomed. Pharmacother..

[182] Navarro-González J.F., Sánchez-Niño M.D., Donate-Correa J., Martín-Núñez E., Ferri C., Pérez-Delgado N., Górriz J.L., Martínez-Castelao A., Ortiz A., Mora-Fernández C. (2018). Effects of Pentoxifylline on Soluble Klotho Concentrations and Renal Tubular Cell Expression in Diabetic Kidney Disease. Diabetes Care.

[183] Lerch C., Shroff R., Wan M., Rees L., Aitkenhead H., Bulut I.K., Thurn D., Bayazit A.K., Niemirska A., Canpolat N. (2018). Effects of nutritional vitamin D supplementation on markers of bone and mineral metabolism in children with chronic kidney disease. Nephrol. Dial. Transplant..

[184] Iyengar A., Kamath N., Reddy H.V., Sharma J., Singhal J., Uthup S., Ekambaram S., Selvam S., Rahn A., Fischer D.-C. (2022). Determining the optimal cholecalciferol dosing regimen in children with CKD: A randomized controlled trial. Nephrol. Dial. Transplant..

[185] Trivedi M.K., Branton A., Trivedi D., Mondal S., Jana S. (2022). Efficacy of a novel proprietary dietary supplement (TRI 360TM) on psychological symptoms and stress-related quality of life in adult subjects: A randomized controlled clinical trial. Front. Psychiatry.

[186] Mahdavi S. (2026). Klotho, Kidneys, and Micronutrient Signaling: A Promising Paradigm for Healthy Aging. Lifestyle Genom..

[187] Ostojic S.M., Hillesund E.R., Øverby N.C., Vik F.N., Medin A.C. (2023). Individual nutrients and serum klotho levels in adults aged 40–79 years. Food Sci. Nutr..

[188] Paquette J.-S., Rhéaume C., Cordeau P., Moulin J.-A., Audet-Walsh E., Blanchette V., Drouin-Chartier J.-P., Toi A.-K., Tremblay A. (2023). The Longevity Protein Klotho: A Promising Tool to Monitor Lifestyle Improvements. Metabolites.

[189] Wu S.E., Chen Y.J., Chen W.L. (2022). Adherence to Mediterranean Diet and Soluble Klotho Level: The Value of Food Synergy in Aging. Nutrients.

[190] Edmonston D., Fuchs M.A.A., Burke E.J., Isakova T., Wolf M. (2024). Chronic Renal Insufficiency Cohort (CRIC) Study Investigators. Klotho and Clinical Outcomes in CKD: Findings from the Chronic Renal Insufficiency Cohort (CRIC) Study. Am. J. Kidney Dis..

[191] Amaro-Gahete F.J., De-La-O A., Jurado-Fasoli L., Gutiérrez Á., Ruiz J.R., Castillo M.J. (2019). Association of physical activity and fitness with S-Klotho plasma levels in middle-aged sedentary adults: The FIT-AGEING study. Maturitas.

[192] Amaro-Gahete F.J., De-La-O A., Jurado-Fasoli L., Espuch-Oliver A., de Haro T., Gutierrez A., Ruiz J.R., Castillo M.J. (2019). Exercise training increases the S-Klotho plasma levels in sedentary middle-aged adults: A randomised controlled trial. The FIT-AGEING study. J. Sports Sci..

[193] Arroyo E., Troutman A.D., Moorthi R.N., Avin K.G., Coggan A.R., Lim K. (2022). Klotho: An Emerging Factor with Ergogenic Potential. Front. Rehabil. Sci..

[194] Arroyo E., Leber C.A., Burney H.N., Narayanan G., Moorthi R., Avin K.G., Warden S.J., Moe S.M., Lim K. (2023). Relationship between klotho and physical function in healthy aging. Sci. Rep..

[195] Corrêa H.d.L., Raab A.T.O., Araújo T.M., Deus L.A., Reis A.L., Honorato F.S., Rodrigues-Silva P.L., Neves R.V.P., Brunetta H.S., Mori M.A.d.S. (2022). A systematic review and meta-analysis demonstrating Klotho as an emerging exerkine. Sci. Rep..

[196] Rippl M., Bidlingmaier M., Deissler L., Martini S., Mueller K., Schluessel S., Schmidmaier R., Schweizer J.R., Tausendfreund O., Welscher L. (2026). Strength but not power training increases soluble alpha klotho levels in pre-frail older adults. Exp. Gerontol..

[197] Saghiv M.S., Sira D.B., Goldhammer E., Sagiv M. (2017). The effects of aerobic and anaerobic exercises on circulating soluble-Klotho and IGF-I in young and elderly adults and in CAD patients. J. Circ. Biomark..

[198] Collins K.A., Ambrosio F., Rogers R.J., Lang W., Schelbert E.B., Davis K.K., Jakicic J.M. (2023). Change in circulating klotho in response to weight loss, with and without exercise, in adults with overweight or obesity. Front. Aging.

[199] Chen J., Zeng Q., Liu L., Li Y., Wang A., Yi Y., Wang Z., Sun W., Zhou W., Ye Y. (2025). The Association Between Weight-Adjusted-Waist Index and S-Klotho Levels in Adults: NHANES 2007–2016. Food Sci. Nutr..

[200] Amaro-Gahete F.J., De-La-O A., Jurado-Fasoli L., Espuch-Oliver A., de Haro T., Gutiérrez Á., Ruiz J.R., Castillo M.J. (2019). Body Composition and S-Klotho Plasma Levels in Middle-Aged Adults: A Cross-Sectional Study. Rejuvenation Res..

